# Breaking a barrier: *In trans vlsE* recombination and genetic manipulation of the native *vlsE* gene of the Lyme disease pathogen

**DOI:** 10.1371/journal.ppat.1012871

**Published:** 2025-01-10

**Authors:** Preeti Singh, Troy Bankhead

**Affiliations:** Department of Veterinary Microbiology and Pathology, Washington State University, Pullman, Washington, United States of America; Texas A&M University, UNITED STATES OF AMERICA

## Abstract

Host-pathogen interactions represent a dynamic evolutionary process, wherein both hosts and pathogens continuously develop complex mechanisms to outmaneuver each other. *Borrelia burgdorferi*, the Lyme disease pathogen, has evolved an intricate antigenic variation mechanism to evade the host immune response, enabling its dissemination, persistence, and pathogenicity. Despite the discovery of this mechanism over two decades ago, the precise processes, genetic elements, and proteins involved in this system remain largely unknown. The *vls* locus, which is the site of antigenic variation, has been notoriously challenging to manipulate genetically due to its highly conserved structural features, even with significant advancements in molecular biology and genetic engineering for this highly segmented pathogen. Our study highlights the pivotal role of plasmid topology in facilitating *in trans* gene recombination. We demonstrate that gene conversion can occur *in trans* when a copy of *vlsE* gene is present on a linear plasmid, contrary to previous observations suggesting a *cis* arrangement is required for *vlsE* recombination. Significantly, employing this *in trans* gene conversion strategy with a linear plasmid, we have, for the first time, achieved targeted genetic mutation of putative *cis*-acting elements in the native *vlsE* gene. This has unveiled a potentially crucial role for the 17 bp direct repeats that flank the central variable cassette region of *vlsE*. Furthermore, we validated the reliability and reproducibility of our mutational approach by successfully inserting stop codons at two distinct sites within the central variable cassette of *vlsE*. Thus, this study presents a significant methodological innovation enabling the direct manipulation of the *vls* locus and lays the groundwork for systematic exploration of specific mutations affecting the mechanism of antigenic variation. As a result, it creates new avenues for research and raises intriguing questions that could guide the development of novel methods to explore host-pathogen interactions of the agent of Lyme disease.

## Author summary

Antigenic variation is one of the key strategies employed by *Borrelia burgdorferi*, the causative agent of the Lyme disease, to evade the host’s adaptive immune response. Despite being recognized for over 25 years, the precise mechanisms underlying this immune evasion system remain poorly understood. This knowledge gap is mainly because the *vls* locus, the site of antigenic variation, has many highly conserved features and secondary structures that complicate genetic manipulation efforts. In this study, for the first time, we successfully introduced mutations in various elements of the *vlsE* gene, a central component of the antigenic variation system. We demonstrate that the plasmid topology significantly influences gene conversion process and identify the 17 bp direct repeat DNA sequences as potentially crucial when a *vlsE* gene copy is present on a separate linear plasmid. Additionally, we validated our method for introducing mutations, proving its effectiveness by creating stop codons at multiple sites within the *vlsE* gene. This new approach provides a robust framework for investigating the impact of specific genetic alterations on the bacterium’s antigenic variability. Such insights are crucial for a deeper understanding of host-pathogen interactions to combat Lyme disease.

## Introduction

Over time microbes have evolved highly advanced mechanisms to adapt and thrive within their environmental niches. Host-pathogen interactions are some of the most dynamic processes: interplay between them has led to the emergence of intricate mechanisms on both sides, each attempting to stay one step ahead of the other. Pathogens have developed different complex strategies to evade hosts’ defenses and maximize transmission efficiency while hosts have evolved sophisticated immune systems to recognize and combat these pathogens. This co-evolutionary process between hosts and pathogens is often described as ‘arms race’, where each side is continuously evolving and adapting to surpass the other in the complex biological competition. *Borrelia* species that cause Lyme disease (Lyme borreliosis) in humans include *Borrelia burgdorferi*, *B*. *afzelii*, *B*. *garinii*, *B*. *mayonii*, *B*. *bavariensis*, *B*. *spielmanii*, *B*. *lusitaniae*, *B*. *bissettii*, and *B*. *valaisiana* [1–9], exemplifying the contemporary concept of emerging infectious diseases [10]. Because of its increasing incidences globally due to a number of demographic and environmental factors including globalization and climate change, it is now the most prevalent tick-borne illness in the Palearctic region of the Northern Hemisphere [11–15]. To successfully complete its enzootic life cycle between *Ixodes* ticks and reservoir hosts, *Borrelia* has evolved numerous strategies to avoid or subvert the host’s immune response.

**Fig 1:**
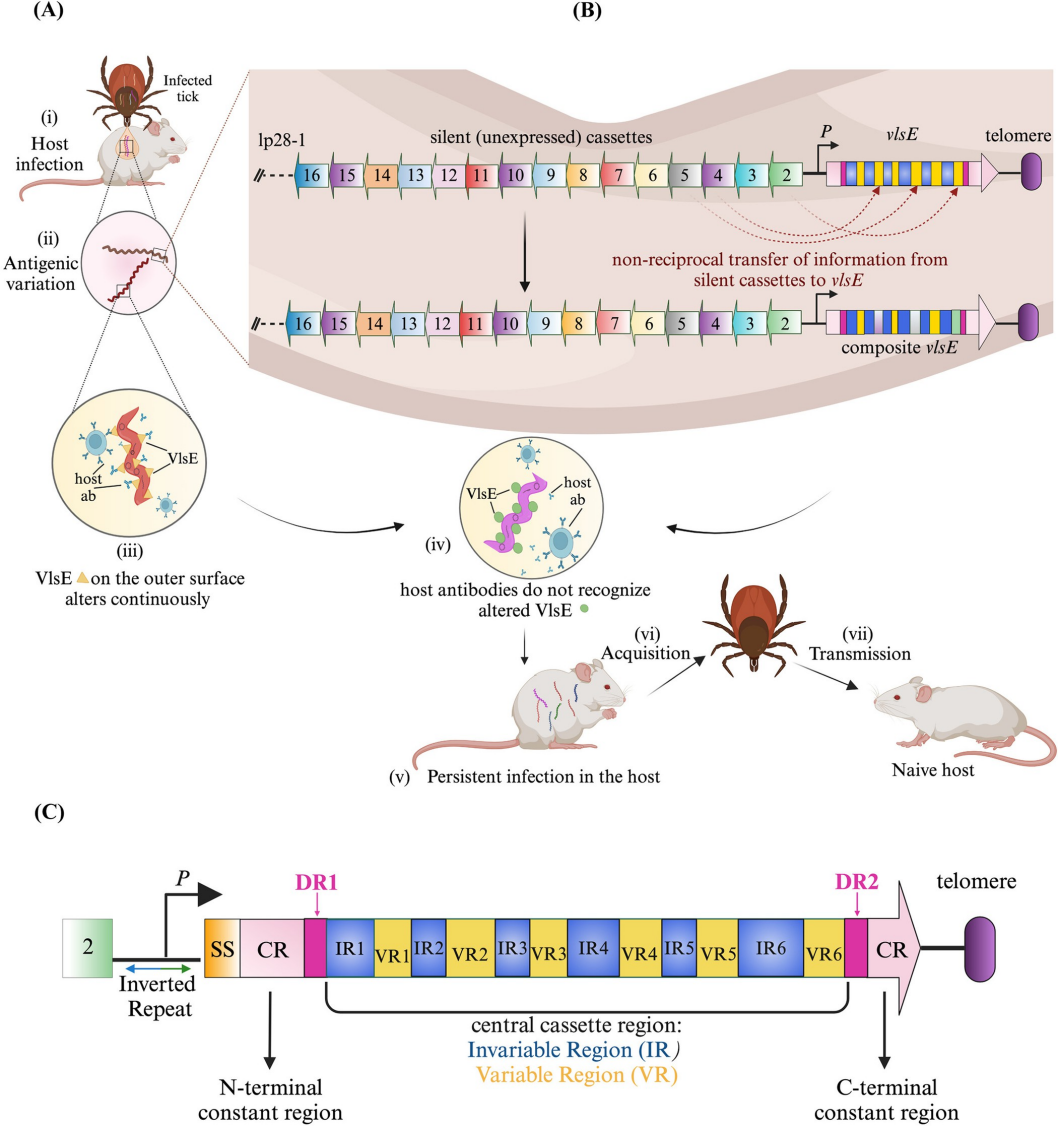
Schematic illustration of the antigenic variation mechanism at the *vls* locus of *B*. *burgdorferi* B31. (A) Critical stages of antigenic variation. When mammalian hosts are infected by *Ixodes* spp. ticks (i), the antigenic variation process is initiated (ii). During this process, the outer surface protein, VlsE, undergoes sequence changes (iii), consequently the variant VlsE would not bind effectively with preformed anti-VlsE antibodies (iv) and thereby enabling the spirochete to establish persistent infection within the host (v). Naïve ticks then acquire the spirochetes from these infected hosts (vi) and are capable of successfully transmitting to subsequent hosts (vii). (B) Molecular mechanism of antigenic variation. The antigenic variation in *B*. *burgdorferi* occurs at the *vls* locus. The *vlsE* expression site, along with its promoter (*P*), is located 82 bp from the covalently closed right end of the linear plasmid lp28-1 (telomere, purple*)*. 15 silent cassettes are arranged in an array, oriented oppositely to the *vlsE* gene. Unknown factor/s within the host trigger a unidirectional, non-reciprocal transfer of genetic information from the silent cassettes to *vlsE* resulting in production of a composite *vlsE* that escapes recognition by the humoral immune response of the host. (C) Primary structure of VlsE. Constant regions (CR, pink) on both- N- and C- terminal ends surround the central cassette region demarcated by 17 bp direct repeats (DR1 and DR2, magenta). The central cassette region is further divided into six invariable regions (IR1-6, blue) and six variable regions (VR1-6, yellow). All the recombination events during mammalian infection occurs in VRs. The intergenic region between *vlsE* and the first silent cassette, 2 (green), contains a 51 bp perfect inverted repeat (shown by bidirectional arrow) that partially overlaps with the promoter (*P*). The signal sequence (SS) of VlsE is shown in orange. The figure was created with BioRender.com.

Antigenic variation, one of the many survival strategies employed by pathogens to escape host immune surveillance, is a widespread phenomenon across different organisms belonging to distant evolutionary lineages. In this game of ‘hide-and-seek’, an infectious microorganism continuously changes its antigenic structure by altering its surface proteins, either genetically or epigenetically, to outsmart the immune response of the host, enabling the pathogen to go undetected to establish persistent infection and be successfully transmitted to the next host (Fig 1A). Various microbes have developed different mechanisms of antigenic variation to evade the host immune response. These include bacteria (*Neisseria* spp. and *Treponema pallidum*) [16–19], protozoa (*Plasmodium falciparum*, *Trypanosoma brucei*, *Giardia lamblia*, and *Babesia bovis*) [20–24], and fungi (*Pneumocystis carinii* and *Candida* spp.) [25–27].

One of the most unique properties of *Borrelia* spp. is its highly segmented genome [28], consisting of numerous circular and linear plasmids along with a linear chromosome [29]. These linear replicons are terminated by covalently closed hairpin ends [30–33]. In the B31 strain of *B*. *burgdorferi*, antigenic variation occurs at the *vls* locus on the linear plasmid, lp28-1 [34,35]. The expressed gene, *vlsE*, is located 82 bp from the right telomere end of the plasmid and encodes a 35 kDa outer surface lipoprotein, VlsE. Upstream of *vlsE* are 15 unexpressed silent cassettes arranged in a contiguous array in the opposite orientation to the *vlsE* gene [34] (Fig 1B). The primary structure of VlsE consists of the central cassette region flanked on both sides by N- and C-terminal constant domains. These domains are demarcated on both sides by 17 bp direct repeats [34] (Fig 1C). Interestingly, identical repeats are also present between most of the silent cassettes. The central cassette region of *vlsE* has 90% sequence identity with the silent cassettes and it is further divided into six variable regions and six invariable regions. Antigenic variation occurs via a non-reciprocal gene conversion mechanism, in which segments within the central cassette region of *vlsE* are replaced by fragments of varying lengths and locations from silent cassettes resulting in composite *vlsE* sequences [36]. Another characteristic feature of the *vls* locus is the presence of a nearly perfect 51 bp inverted repeat in the intergenic region between *vlsE* and first silent cassette [37]. Previous studies have shown that antigenic variation occurs only during host infection [35,38]. This variation can be detected as early as four days post-infection, and by day 14, the presence of parental clones is minimal or nonexistent [35,39]. Furthermore, the expression of VlsE is upregulated 32-fold during murine infection relative to *in vitro* conditions [40].

Since its discovery in 1997 [34,41], unraveling the mechanistic intricacies of antigenic variation in *Borrelia* has proven to be a significant scientific challenge. The complexity arises from the limitations of numerous molecular biology techniques in effectively probing this genomic locus due to several factors, including its sub-telomeric position, the repetitive nature of the silent cassettes, the high sequence identity among them, the presence of inverted repeats, and the formation of potential secondary structures. These factors collectively render cloning extremely difficult and unstable in *Escherichia coli* [41]. Furthermore, a straightforward PCR analysis of *vlsE*, along with its native promoter, often yields misleading results due to the presence of numerous false priming sites and the difficulty in achieving the correct amplification products. These issues are further complicated by the fact that gene conversion does not occur *in vitro* or in ticks [42]. Consequently, studies must resort to time-consuming murine infection experiments to investigate recombinational events [41]. Currently, information available about the factor(s) that triggers the gene conversion mechanism during host infection is lacking [43] and only limited data are available about the proteins involved in *vlsE* recombination [44–47]. Hence, the exploration of the *vls* locus through genetic analysis has been restricted to a few studies. Initial findings demonstrated that the absence of lp28-1 reduced infectivity, establishing its essential requirement for the full infectivity in the host [48,49]. A subsequent study confirmed the crucial role of the *vls* locus in host persistence by deleting it from the right end of lp28-1 via telomere resolvase-mediated targeted deletion [50]. A similar approach showed that spirochetes expressing a static *vlsE* (non-variable) were capable of initial infection but were cleared by three weeks post-infection, indicating the necessity of *vlsE* variability for long-term persistence in immunocompetent mice [51,52]. *vlsE*-deficient mutants are fully infectious in immunodeficient (SCID) mice, indicating *vlsE* expression and sequence variation are not required for the bacterium’s survival and confirming the primary role of the *vls* system in immune evasion [50,52–54]. Furthermore, non*-vls* genes residing on lp28-1 were found to be non-essential for persistence in the mammalian host [55]. However, these studies predominantly focused on the *vls* locus in its entirety. To address these gaps, Castellanos et al. [56] developed a mini-*vls* system which allowed genetic manipulation of 51 bp inverted repeat. Their findings suggested that plasmid topology carrying the *vls* system is not important for gene conversion and inverted repeat is required only when *vls* is in a circular form, not in linear form. However, this system was based on an artificial plasmid backbone and may not accurately represent the wild-type context [56]. Therefore, questions still remain regarding the specific role(s), if any, of conserved elements in *vlsE* and whether a *cis* arrangement of *vlsE* and silent cassettes on lp28-1 is necessary for gene conversion.

In this study, we show that gene conversion can occur *in trans* when a *vlsE* gene copy is located on a linear shuttle plasmid. Utilizing this approach, we demonstrate a potentially critical role of the 17 bp direct repeats in the genetic recombination processes that underpin antigenic variation. Given the multipartite nature of the genome of *B*. *burgdorferi*, our findings highlight the integral role of plasmid topology in the gene conversion mechanism. Most importantly, we also introduce a method for the direct mutagenesis of several components of the native *vlsE* gene on the lp28-1 plasmid in *B*. *burgdorferi*, marking a step forward in the field of genetic engineering of this enigmatic pathogen. This technique facilitates the precise introduction of mutations into the *vlsE* gene, a key element in the pathogen’s antigenic variation system. Moreover, to further establish the strength and reliability of our approach, we expanded its application successfully by introducing a stop codon in two separate invariable regions within the central cassette of *vlsE*. Thus, mutating these three distinct sites within the *vlsE* gene demonstrates the robustness, precision, and versatility of our method. This approach addresses previous limitations in the genetic studies of this locus and creates new opportunities for investigating the molecular mechanisms governing antigenic variation in *B*. *burgdorferi*.

## Results

### Gene conversion of *vlsE* can occur *in trans*

**Fig 2:**
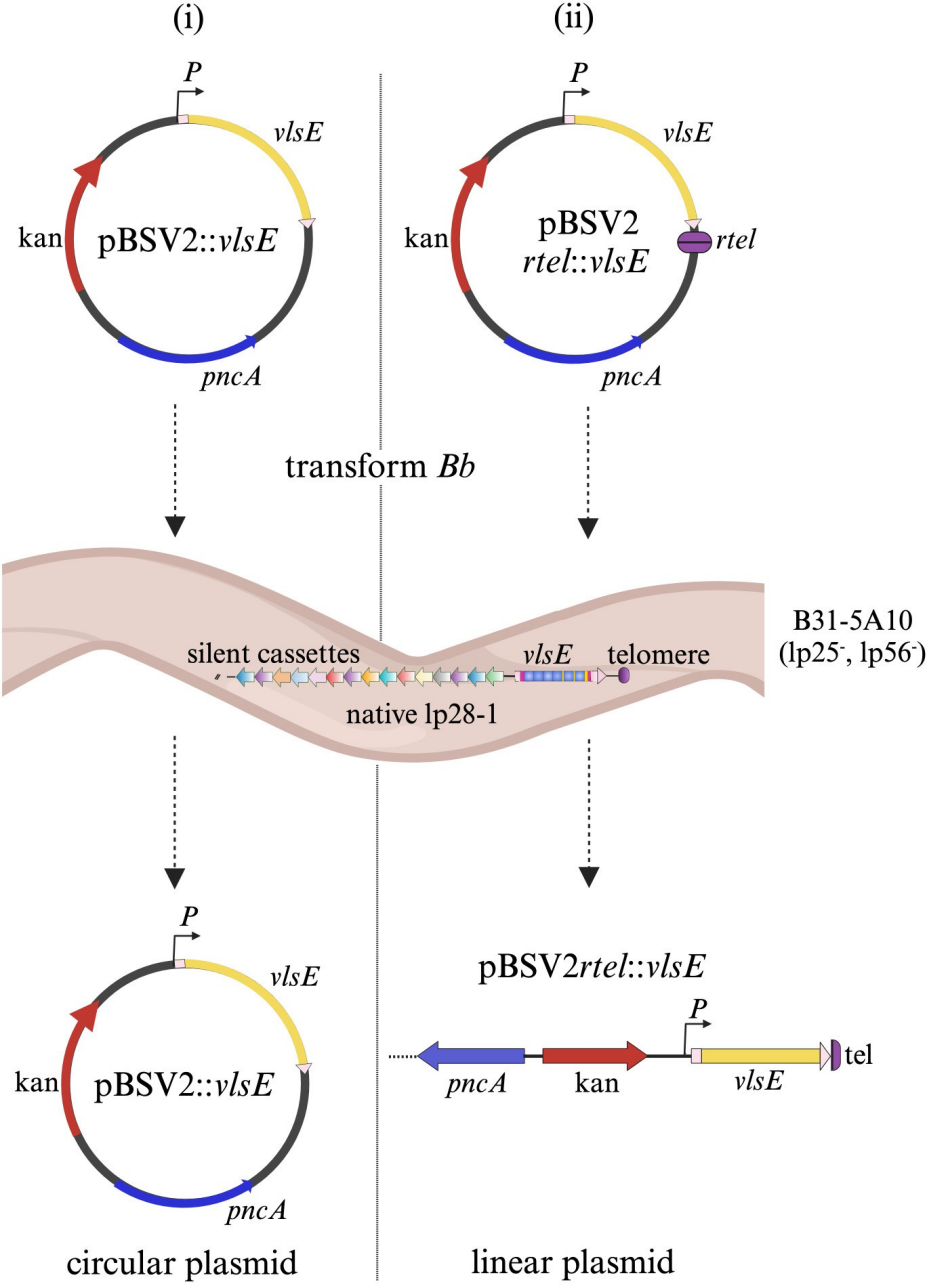
Gene conversion of *vlsE* can occur *in trans*. Schematic representation of (i) circular (pBSV2::*vlsE*) and (ii) linear *vlsE (*pBSV2*rtel*::*vlsE)* plasmid constructs. *vlsE* along with its native promoter (P) (yellow) was cloned into the *B*. *burgdorferi- E*. *coli* shuttle vector pBSV2 containing a kanamycin resistance marker (red) and the *pncA* gene (blue). Plasmid pBSV2::*vlsE* retained its circular form when transformed into *B*. *burgdorferi* 5A10 cells. To linearize the plasmid following transformation into *B*. *burgdorferi*, a replicated telomere (*rtel*, purple*)* was cloned into the construct, creating pBSV2*rtel*::*vlsE* having single telomere at each end. Silent cassettes present on lp28-1 in 5A10 serve as ‘donor’ sequences for gene conversion. The figure was created with BioRender.com.

In all Lyme disease species of *Borrelia*, the *vls* locus is always found near the telomere end of a linear plasmid [2,57–59], while many other bacterial antigenic variation systems are located on circular molecules [60]. Because of this, we explored the potential importance of plasmid topology in gene conversion. A previous study demonstrated that *vlsE* gene conversion did not occur with a *trans* copy of *vlsE* during infection, suggesting a possible *cis* requirement for the *vlsE* recombination mechanism [52]. However, the *trans* copy of *vlsE* in that study was carried on a circular shuttle vector, as opposed to the linear lp28-1 in which *vlsE* normally resides. We investigated the ability of a linear *trans* copy of *vlsE* to undergo recombination and determine whether spirochetes harboring this linear plasmid expressing VlsE can persistently infect mice. The *vlsE* gene with its native promoter was synthesized commercially and cloned into the pBSV2 shuttle vector containing *pncA*, which encodes a nicotinamidase located on lp25, a gene essential for mouse infection in the absence of lp25 [54]. A replicated telomere (*rtel*) from lp28-1 was also added, which allows for conversion into a linear form by the endogenous telomere resolvase, ResT, once introduced into *Borrelia* cells [61–63] (Fig 2). The plasmids were then individually transformed into *B*. *burgdorferi* 5A10 cells. This strain contains native lp28-1 but lacks lp25 and lp56 plasmids, which harbor restriction/modification genes, allowing for an increase in transformation efficiency of these *B*. *burgdorferi* cells [64,65]. Due to harboring both lp28-1 and the *vlsE* gene copy, the silent cassettes present on lp28-1 could potentially serve as ‘donor’ sequences for gene conversion of *vlsE* on the linear shuttle vector. Prior to infection in mice, increased expression levels (due to both *vlsE* copies) and surface localization of VlsE in transformant clones were verified by treating with or without proteinase K and subjecting to Western blot analysis. Groups of six C3H/HeN mice were then infected with *B*. *burgdorferi* B31-5A10 clones harboring a *vlsE* copy on either a circular or linear shuttle plasmid. A group of mice was infected with the wild type B31-A3 clone as a control. The results showed that all mice initially infected with spirochetes harboring a *vlsE* copy on a circular plasmid were eventually cleared by four weeks post-infection, while four out of six mice infected with spirochetes harboring the *vlsE* copy on a linear plasmid were not cleared of infection by four weeks (Table 1). Next, we assessed whether the *vlsE* gene copy on the shuttle vector could undergo gene conversion. Total DNA was extracted from spirochetes recovered from bladder tissues at week two and were subjected to field-inversion gel electrophoresis (FIGE). This technique was employed to spatially separate the native *vlsE* on the lp28-1 plasmid (28 kb) from the linear shuttle vector gene copy (8.6 kb), ensuring their distinct positioning and mitigating the risk of cross-contamination in subsequent analyses. Individual shuttle plasmid bands were excised from the gel and purified DNA was used as a template to amplify central variable cassette of *vlsE* using primers P243 and P244 (S1 Table). The amplicons were TOPO cloned and sequenced using M13 primers. DNA sequencing analysis of 16 clones showed that all sequences in the circular shuttle plasmid group were nonvariant, with no changes observed across the variable regions. This finding suggests that the *vlsE* copy on the circular plasmid did not undergo recombination, and clearance was likely due to the expression of non-variable VlsE, which was targeted by host antibodies. In contrast, in the linear shuttle plasmid group, 10 out of 12 clones were variant, exhibiting changes in the variable regions indicating that the linear plasmid *vlsE* copy had undergone gene conversion. Thus, the proportions of nonvariant to variant clones were 100 percent nonvariant in the circular plasmid group and 83 percent variant in the linear plasmid group (Table 1).

**Table 1:** Results demonstrating the difference in the ability of *vlsE* copy harbored on a circular or linear plasmid shuttle vector in B31-5A10 strain to persistently infect mice and undergo *vlsE* gene conversion.

B31 clone	Expression of introduced *vlsE* copy	C3H mice at week four p.i.	*vlsE* copy gene conversion
A3 (WT)	N/A	6/6	N/A
5A10/pBSV2::*vlsE* (circular)	+	0/6	0/16^a^
5A10/pBSV2*rtel*::*vlsE* (linear)	+	4/6	10/12

^a^ Indicates the number of TOPO clones that showed variation out of the total number of clones that were screened for changes in the variable region of the indicated *vlsE* gene copy.

### Generation of a *B*. *burgdorferi* clone harboring mutations in direct repeat regions

**Fig 3:**
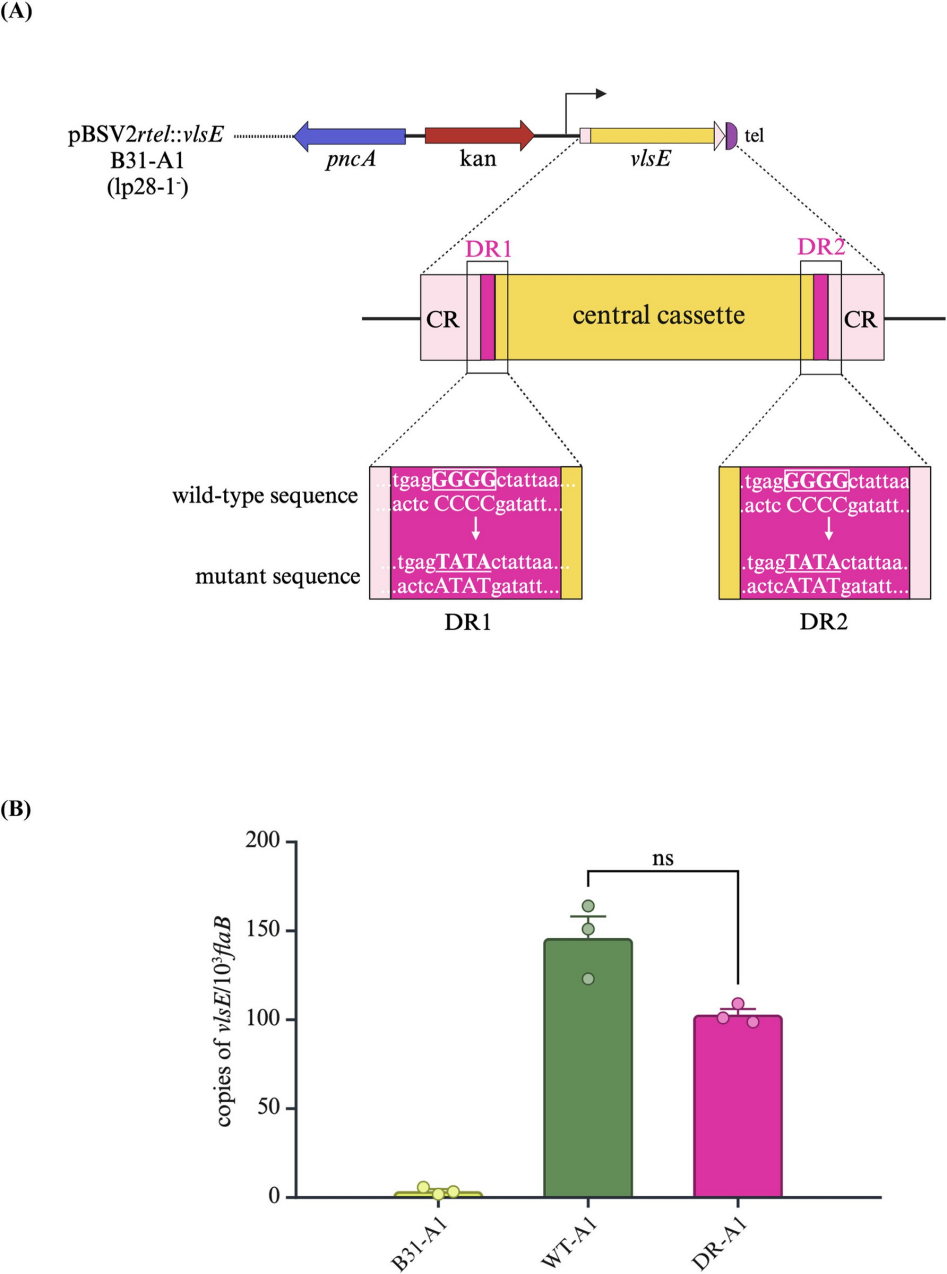
Construction and analysis of mutations in direct repeats (DR) regions of *vlsE*. (A) Schematic drawing of mutations generated in direct repeat (DR) regions (magenta) flanking the central cassette of *vlsE* (yellow) on the linear shuttle vector pBSV2*rtel*::*vlsE*. A stretch of guanine bases in the wild-type sequence (boxed) were replaced with adenine and thymine bases (underlined) in the mutant sequence in both DR1 and DR2 regions as illustrated in the magnified box. Constant regions (CR, pink) are marked on both sides of the central cassette. Plasmids carrying either wild-type or the mutated copy of *vlsE* were each transformed into *B*. *burgdorferi* A1 and 5A10 strains. (B) qRT-PCR assessment of changes in the transcription level of *vlsE*. Total RNA was extracted from *in vitro* grown wild-type A1 strain and A1 harboring either wild type or mutated *vlsE* copy on a linear plasmid, converted to cDNA, and evaluated through ddPCR wherein *vlsE* transcripts levels were normalized to 1000 copies of *flaB* using a TaqMan assay. Values were expressed as an average of triplicates ± standard error mean. No significant (ns) difference in the *vlsE* transcript levels were observed between wild type and DR mutant strains using Student’s paired *t*-test (*p*>0.05). The figure was created with BioRender.com.

An earlier study suggested that the 17 bp direct repeat (DR) sequences flanking the central variable cassette of *vlsE* may have a role in gene recombination, but direct mutational evidence for its role *in vivo* was lacking [66]. Because our previous experiment demonstrated that the *vlsE* copy on a linear shuttle vector can undergo gene conversion *in trans*, we generated mutations in both DR regions flanking the central cassette of the *vlsE* gene copy. To accomplish this, mutations in the 5’ and 3’ 17 bp DR (DR1 and DR2, respectively) were generated by replacing a stretch of four nucleotides of guanine with adenine and thymine bases (GGGG-to-TATA; Fig 3A). Once the mutations were confirmed, plasmid pBSV2*rtel*::*vlsE*:DR was transformed into the *B*. *burgdorferi* B31-A1 strain. As a control, a wild type gene copy of *vlsE* on the shuttle plasmid (pBSV2*rtel*::*vlsE*) was also transformed into B31-A1. B31-A1 contains all the plasmids required for persistent infection except for the *vls*-resident plasmid, lp28-1 [67]. We next assessed if the DR mutations have any effect in the transcription of *vlsE*. Total RNA was isolated from *in vitro*-grown cultures of B31-A1 spirochetes harboring either a wild type or mutant *vlsE* copy on the shuttle vector, converted into cDNA, and subjected to qRT-PCR using droplet digital PCR (ddPCR). Analysis of *vlsE* transcript levels indicated no statistically significant difference in the transcript levels of *vlsE* between the wild type and DR mutant strains, indicating DR mutation has no effect on *vlsE* transcription under *in vitro* conditions (Fig 3B).

To determine whether the GGGG-to-TATA mutation, leading to an amino acid substitution from Gly-Ala to Tyr-Thr in both DR regions, affects VlsE protein expression levels and/or surface localization in the mutant strain, we performed Western blot analysis with or without proteinase K digestion. Following this treatment, samples were immunoblotted with anti-VlsE and anti-FlaB antibodies. The results showed similar VlsE protein expression in both the wild type and DR mutant strains, indicating that the mutation did not impact overall protein levels (S1 Fig). Additionally, VlsE was confirmed to be surface-exposed in the DR mutant strain, consistent with its localization in the wild type strain.

One additional note of importance is that both the wild type and DR mutant *vlsE* gene copies on the shuttle plasmids harbored mutations in the promoter region. These alterations involved a T-to-C change at the –38 position (adjacent to the –35 box) and an A-to-G substitution, which resulted in an amino acid change from glutamic acid to lysine at the third position of the signal peptide region. We chose these mutations to temper the expression of the *vlsE* gene copies to prevent excessive overall VlsE production in the clones due to the additional presence of the native *vlsE* gene on lp28-1.

To evaluate the impact of these mutations on VlsE protein production, we compared protein levels between a mutant promoter strain and a wild type promoter strain. Western blot analysis showed the marked reduction in VlsE expression in the wild type gene copy strain harboring a mutated promoter relative to the wild type gene copy strain with a wild type promoter (S2 Fig).

### Mutations in DR regions inhibit *in trans vlsE* gene conversion

**Fig 4:**
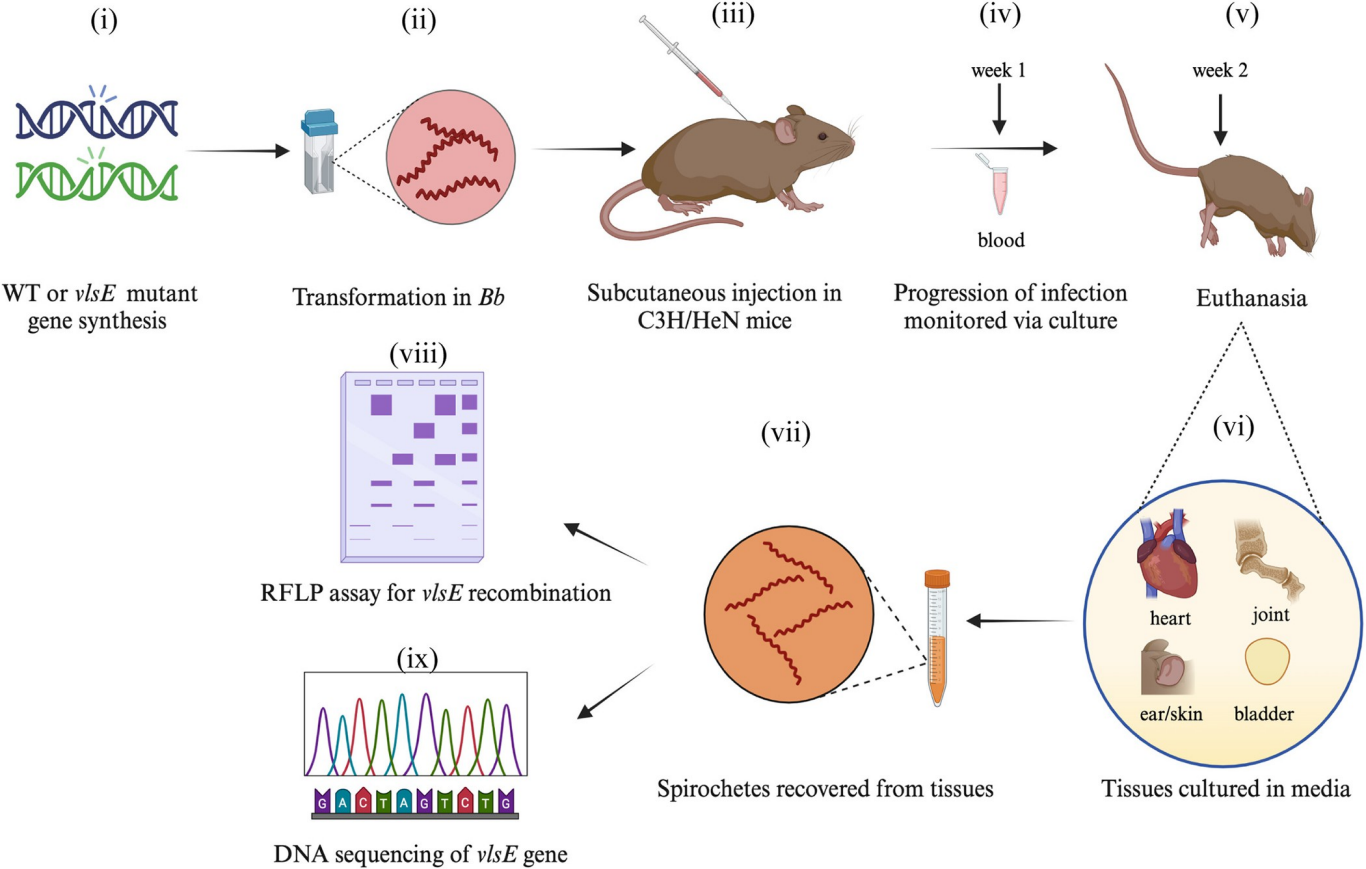
Animal experimental design. Schematic representation of the experimental strategy adopted to assay for *vlsE* recombination during *Bb* infection of mice. (i) Shuttle plasmids containing either the wild-type or mutated copy of *vlsE* were constructed, (ii) *B*. *burgdorferi* 5A10 strain was transformed, (iii) groups of three C3H/HeN mice each were infected subcutaneously via needle inoculation, (iv) progress of infection was monitored for 2 weeks by culturing blood at week 1, followed by (v) euthanasia of mice at week 2, (vi) portions of skin, bladder, heart and joint were harvested and cultured in BSK-II media. After a week (vii) total DNA was isolated from the recovered spirochetes and subjected to (viii) PCR-RFLP assay and (ix) DNA sequencing of the variable region of *vlsE* gene to assess *vlsE* recombination. The figure was created with BioRender.com.

To investigate the role of the DRs in infectivity, persistence, and gene conversion during host infection, wild type or mutant *vlsE* gene copies residing on the pBSV2*rtel* shuttle plasmids were transformed into B31-5A10 cells. The clones were validated by Southern blot analysis (S3 Fig) and plasmid profiles were also confirmed. The experimental animal infection strategy is outlined in Fig 4. Briefly, groups of three C3H/HeN mice were infected with B31-5A10 transformed with constructs containing either wild type or DR mutated *vlsE* sequences, along with B31-A3 as a control. Progression of the infection was followed for two weeks: at week 1, blood was cultured in BSK-II media, then at week 2, mice were euthanized, and portions of skin, bladder, heart, and joints were harvested and cultured into media for the recovery of the spirochetes. The experiment was performed in triplicate. At week 2 post-infection, all nine mice in the A3 control group tested positive for spirochetes in all 36 sites tested. In contrast, spirochetes were successfully cultured from only 15 out of 32 sites in mice infected with wild type strain (pBSV2*rtel*::*vlsE*). In the group infected with the DR mutant strain, spirochetes were recovered from 13 of the 36 tested sites, as summarized in Table 2. However, this difference was not statistically significant using Fisher’s Exact test (*p*>0.05).

**Table 2:** Effect of DR mutations present in B31-5A10 strain on *B*. *burgdorferi* infection in C3H/HeN mice.

	B31 clone^a^	Blood^b^	Ear	Bladder	Heart	Joint	Total sites^c^	Total mice^d^
**Wk 1**	A3 (WT)	8/9						
	SV::*vlsE*:WT^e^	3/8						
	SV::*vlsE*:DR^**f**^	1/9						
**Wk 2**	A3 (WT)		9/9	9/9	9/9	9/9	36/36	9/9
	SV::*vlsE*:WT		5/8	5/8	1/8	4/8	15/32	5/8^g^
	SV::*vlsE*:DR		4/9	5/9	1/9	3/9	13/36	5/9

a. Data represent the combined results of three replicate experiments and each experiment contained three mice per group.

b. Values listed correspond to number of cultures positive/number tested at either blood, ear, heart, bladder or joint tissues.

c. Number of positive tissue sites/number tested.

d. Number of culture positive mice /total mice.

e. B31-5A10/pBSV2*rtel*::*vlsE–*linear shuttle vector (SV) containing wild type *vlsE* in *Bb* 5A10 strain.

f. B31-5A10/pBSV2*rtel*::*vlsE*:DR–linear shuttle vector (SV) containing DR mutant *vlsE* in *Bb* 5A10 strain.

g. one mouse did not get infected as none of the tissues tested were positive at both time points, hence it was not included in data analyses.

**Fig 5:**
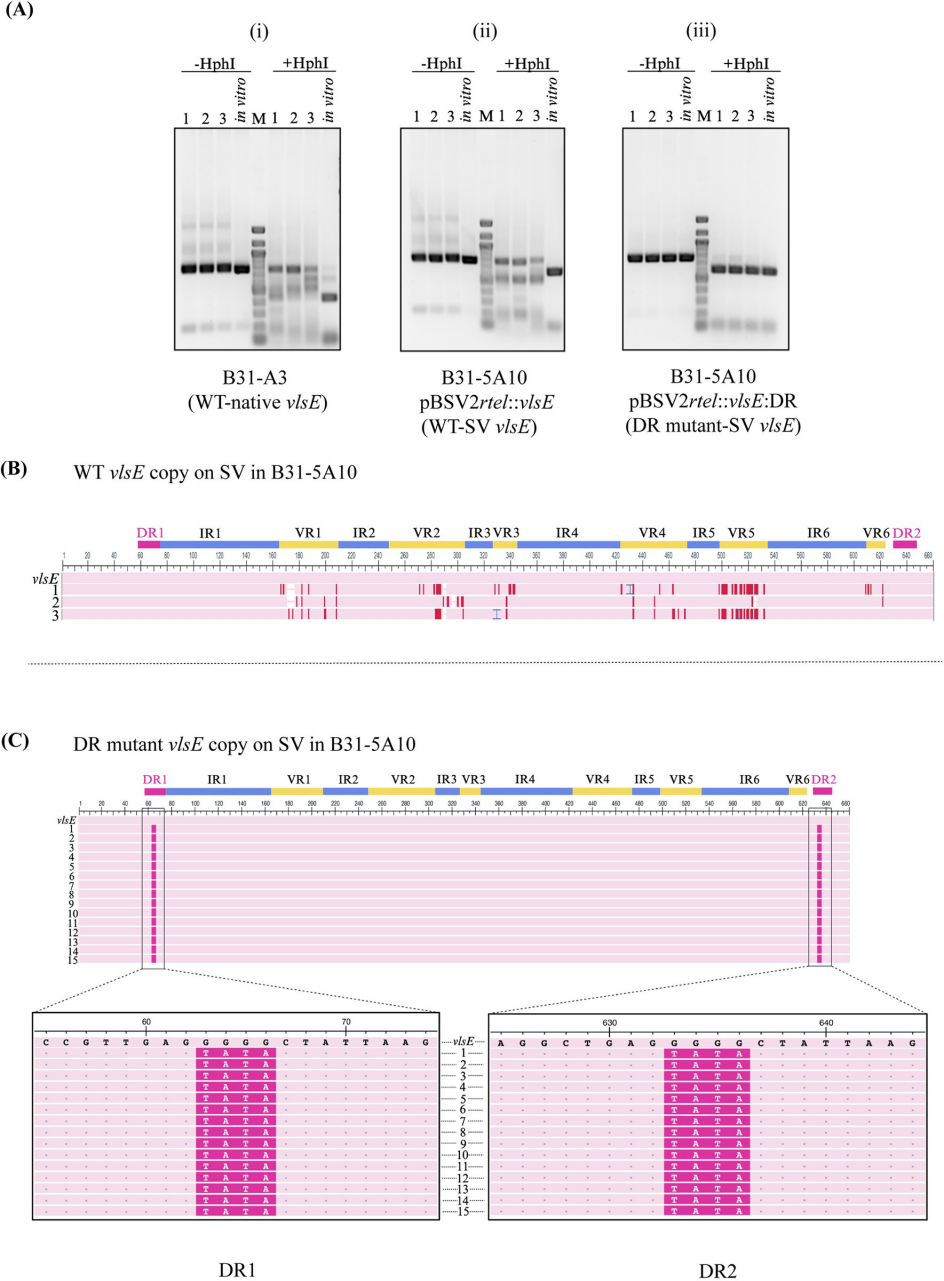
Mutations in direct repeat (DR) regions inhibit *in trans* gene conversion. Three groups of C3H mice (3 animals/group) were infected via needle inoculation with *Bb* 5A10 strain harboring either the wild type or DR mutant copy of *vlsE* residing on the linear shuttle plasmid pBSV2*rtel*::*vlsE*. Wild type B31*-* A3 was also used to infect mice as a control. Total DNA was extracted from the recovered spirochetes from the bladder tissue at week 2 p.i. and subjected to field-inversion gel electrophoresis. The shuttle vectors harboring *vlsE* copy (8.6 kb) were gel excised and used as template for amplification of the central cassette region of *vlsE*. For B31-A3 control mice, the *vlsE* variable cassette was amplified directly from total DNA containing native lp28-1 plasmid. (A) PCR-RFLP analysis. The amplified *vlsE* genes were analyzed undigested or digested with HphI both *in vitro* and after inoculation in C3H mice as indicated. Results showed that both WT controls—(i) A3 and (ii) WT *vlsE* copy on SV displayed a banding pattern due to introduction of additional and/or removal of HphI sites indicative of *vlsE* gene conversion while (iii) DR mutant *vlsE* copy on SV did not show any recombination. SV, shuttle vector; 1, 2, 3 corresponds to mouse 1, mouse 2 and mouse 3 respectively; M, 100 bp marker. (B) Multiple sequence alignment of WT copy of *vlsE* on the linear shuttle vector. DNA sequencing was performed on PCR product amplified from the wild type *vlsE* copy of the linear shuttle vector, as described in (A). These amplicons were TOPO cloned and sequenced using M13 primers. The alignment results revealed changes across the six variable regions (VR1-6) of *vlsE*, indicating recombination events in spirochete populations from all three mice. (C) Multiple sequence alignment of *vlsE* copy on SV in the DR mutant group. Limiting dilution plating of the spirochetes recovered from each mouse was performed and 15 clones (five from each mouse) were randomly picked for sequencing and processed as above. All the 15 clones showed the mutant sequence in both DR1 and DR2 regions, highlighted in magnified boxes while there were no changes in any of the variable regions implying that the DR mutations in SV inhibit gene conversion *in trans*. Positions where the sequences match the anchor sequence (*vlsE*) are colored in pink (and as grey dots in magnified box), while positions that contain mismatches are colored in red. Gaps are indicated by a pink line in white space while insertions relative to the anchor sequence are indicated by a blue bracket. Mutant sequences in DR1 and DR2 are shown in magenta, and the wild type sequence is shown in black in the magnified box. The alignment ruler spans the positions 1–660, starting at the *vlsE* sequence where primer P243 binds the N-terminal constant region and ending at 16 bp of the C-terminal constant region. To highlight the specific regions where changes occurred during recombination events, a color-coded bar was added: magenta for direct repeat regions (DR1, DR2), blue for invariable regions (IR1-6), and yellow for variable regions (VR1-6). Numbers on the left are the clones recovered from the mice and used in sequencing.

Next, we assessed whether the DR mutant clones could undergo gene conversion compared to the wild type. Total DNA extracted from spirochetes recovered from bladder tissues was subjected to FIGE and PCR as described above. To detect whether *vlsE* recombination occurred during murine infection, we applied a two-pronged approach (Fig 4). A previously established method of PCR-restriction fragment length polymorphism (RFLP) was used [68]. In this assay, PCR products corresponding to the variable region of *vlsE* using primers P243 and P244 (S1 Table), were subjected to restriction analysis to identify new HphI restriction endonuclease cleavage sites introduced and/or removed by recombination at *vlsE*. This assay offers solely a qualitative indication of recombination within the sample but lacks sensitivity and quantitative measurement capabilities. Consequently, a secondary method was employed involving DNA sequencing of the *vlsE* amplicons. Because gene conversion occurs only during mammalian infection, *vlsE* variable cassette amplified from *in vitro* grown culture was used as a negative control. For B31-A3 control mice, the *vlsE* variable cassette was amplified directly from total DNA, restriction digestion of PCR amplicons was performed using the HphI enzyme, and products were electrophoresed on 2% agarose gel for RFLP analysis. As shown in Fig 5A, recombination at *vlsE* occurred efficiently in both wild type groups and showed a banding pattern indicative of *vlsE* gene conversion (i and ii). Interestingly, the DR mutant did not display any *vlsE* recombination (Fig 5A-iii). The experiment was repeated two more times and resulted in the same outcome.

To verify *vlsE* gene conversion from the RFLP assays, DNA sequencing of *vlsE* amplicons was conducted. PCR products of the central cassette region of *vlsE* gene copies from the infected mice recovery pool were TOPO cloned and sequenced using M13 primers. Wild type *vlsE* showed changes throughout the six variable regions (Fig 5B). In contrast, sequencing of *vlsE* from the DR mutant group did not show any sequence changes in the variable regions, indicating a lack of gene conversion in the mutated *vlsE* gene copy. Because spirochetes recovered from each mouse represented a heterogeneous population, we also conducted limiting dilution plating of the clones. A total of 15 clones (five from each mouse) were randomly picked and processed individually as before. Again, DNA sequencing revealed the presence of mutation in both DR regions but did not show any changes in the variable regions of the central cassette of the *vlsE* copy on shuttle vector, thus confirming that mutations in the DR regions inhibit gene conversion of *vlsE in trans* (Fig 5C). Complete sequences are available as supplementary data (S4 and S5 Figs).

### Discovery of method to introduce mutations into the native *vlsE* gene on lp28-1

**Fig 6:**
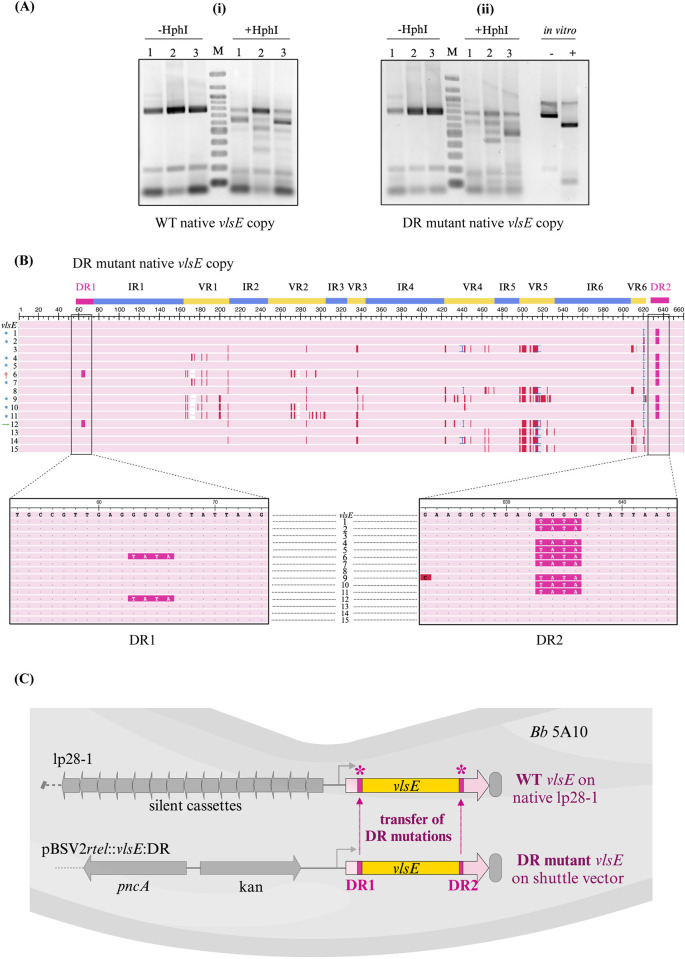
Introduction of mutations into native *vlsE* on lp28-1. (A) PCR-RFLP analysis of native *vlsE* on lp28-1. The experiment was conducted as outlined in Fig 5. Native lp28-1 (28 kb) was gel excised and used as the template for PCR followed by RFLP assay. Both the (i) WT and (ii) DR mutant exhibited banding patterns that suggests *vlsE* gene conversion, indicative of recombination events. *vlsE* amplicon from *in vitro* grown culture was used as a negative control as before.1, 2, 3 corresponds to mouse 1, mouse 2 and mouse 3 respectively; M, 100 bp marker. (B) Multiple sequence alignment of native *vlsE* on lp28-1 in DR mutant. 15 clones from three mice were randomly selected and sequenced as described above. One clone showed incorporation of mutations into the native *vlsE* on lp28-1 in both DR regions (†) from the shuttle vector copy while several more clones were found to incorporate mutations either in the DR1 (green arrow) or DR2 (*) region only. Magnified boxes indicate detailed sequence in DR1 and DR2 regions in all clones. Color coding is same as described in Fig 5. (C) A schematic illustration depicting the transfer or “movement” of mutations within DR1 and DR2 regions from the *vlsE* gene copy carried on the shuttle vector (pBSV2*rtel*::*vlsE*:DR) to the *vlsE* gene copy residing on the native lp28-1 plasmid in the 5A10 strain of *B*. *burgdorferi*. The recipient *vlsE* gene copy on lp28-1 is marked with asterisks for clarity.

We next investigated alterations in the sequence of the native *vlsE* located on lp28-1 in both the wild type and DR mutant groups. RFLP analysis revealed the typical banding profile resulting from HphI digestion of the central variable cassette of the native *vlsE* gene (Fig 6A) as it was anticipated that gene conversion had occurred in the native *vlsE* gene sequence.

The most unexpected findings emerged from the analysis of DNA sequencing data. Following TOPO cloning and subsequent sequencing, we observed the expected sequence variations across all six variable regions of the native *vlsE* gene, corresponding to the RFLP profile. However, upon examining the native *vlsE* gene on the lp28-1 plasmid in the DR mutant group, we discovered that some clones exhibited DR- specific mutations. To investigate this further, we conducted limiting dilution plating of the recovered clones, randomly selecting 15 clones (five from each mouse) for sequencing. The results were revealing: ten clones carried the TATA mutation (replacing GGGG) in either DR1, DR2 or both DRs regions. Specifically, eight clones had this mutation only in DR2 region, while one clone each had the mutation in DR1 alone and in both DR regions (Figs 6B and S6). These results suggest that the *vlsE* sequence on the shuttle vector served as a template for the native *vlsE* gene, transferring and incorporating these sequences into the native *vlsE* gene. This sequence “movement” from the shuttle vector to the native *vlsE* directly modified the native gene. This process is schematically illustrated in Fig 6C. The implications of these results are profound: for the first time, mutations were directly introduced into the *vlsE* gene within the native lp28-1. Previously, technical limitations restricted the ability to introduce such mutations, limiting functional analyses of *vlsE* and the broader understanding of its role in immune evasion. This represents a significant leap forward in our ability to manipulate and understand the genetic underpinnings of antigenic variation. Table 3 summarizes the observations about the clones harboring DR mutations in either native or shuttle vector *vlsE* copy in B31-5A10 strain and their correlation with gene conversion.

**Table 3:** Summary of DR mutations and gene conversion association in B31-5A10 strain.

Mutation Location	DR1^a^ Mutation	DR2^b^ Mutation	Gene^c^ Conversion in Native *vlsE*	Gene^d^ Conversion in SV^e^ *vlsE*
Native *vlsE* with DR1	+	-	+	N/A
Native *vlsE* with DR2	-	+	+	N/A
Native *vlsE* with DR1/DR2	+	+	+	N/A
SV *vlsE* with DR1/DR2	+	+	N/A	-

^a^ Mutation in DR1 region

^b^ Mutation in DR2 region

^c^ Gene conversion occurs in the native *vlsE* gene copy on lp28-1

^d^ Gene conversion occurs in the *vlsE* gene copy on the shuttle vector

^e^ No clones were detected having a single DR mutation in the *vlsE* gene copy on the shuttle vector.

The subsequent aim was to further verify the mutations in native *vlsE* on lp28-1 after eliminating the shuttle vector copy. We investigated this possibility by applying the strategy of plasmid incompatibility. To enable the displacement of this plasmid, we introduced a different shuttle vector, pBSV2G, which incorporates a gentamicin resistance marker. The *pncA* gene was cloned as previously described, and this construct lacked any *vlsE* gene copy. This new plasmid was then transformed into clones recovered from the mice, specifically those harboring mutations in the DR2 region of the native *vlsE* gene, as these clones represented the predominant population. The transformants were selected by growing them in gentamicin-containing media, ensuring that only those with the new plasmid would survive. DNA was extracted from these transformants and subjected to PCR and Southern hybridization using probes specific for *vlsE* and kanamycin. Our results showed that all of the transformed clones had successfully replaced the original shuttle vector (pBSV2*rtel*::*vlsE)* with the new plasmid (pBSV2G:*pncA)*. Importantly, this meant that each of the clones now only had one copy of *vlsE* gene on the native lp28-1 plasmid. Among the clones analyzed, nearly 25 percent still harbored the DR2 mutation in native *vlsE* gene. This finding further confirmed that the mutation had been successfully incorporated into the native *vlsE* on lp28-1, validating our previous observations and demonstrating the effectiveness of our approach for introducing and verifying mutations directly in the *vlsE* gene (S7 Fig).

### Incorporation of mutations into the central variable cassette of *vlsE* on lp28-1

**Fig 7:**
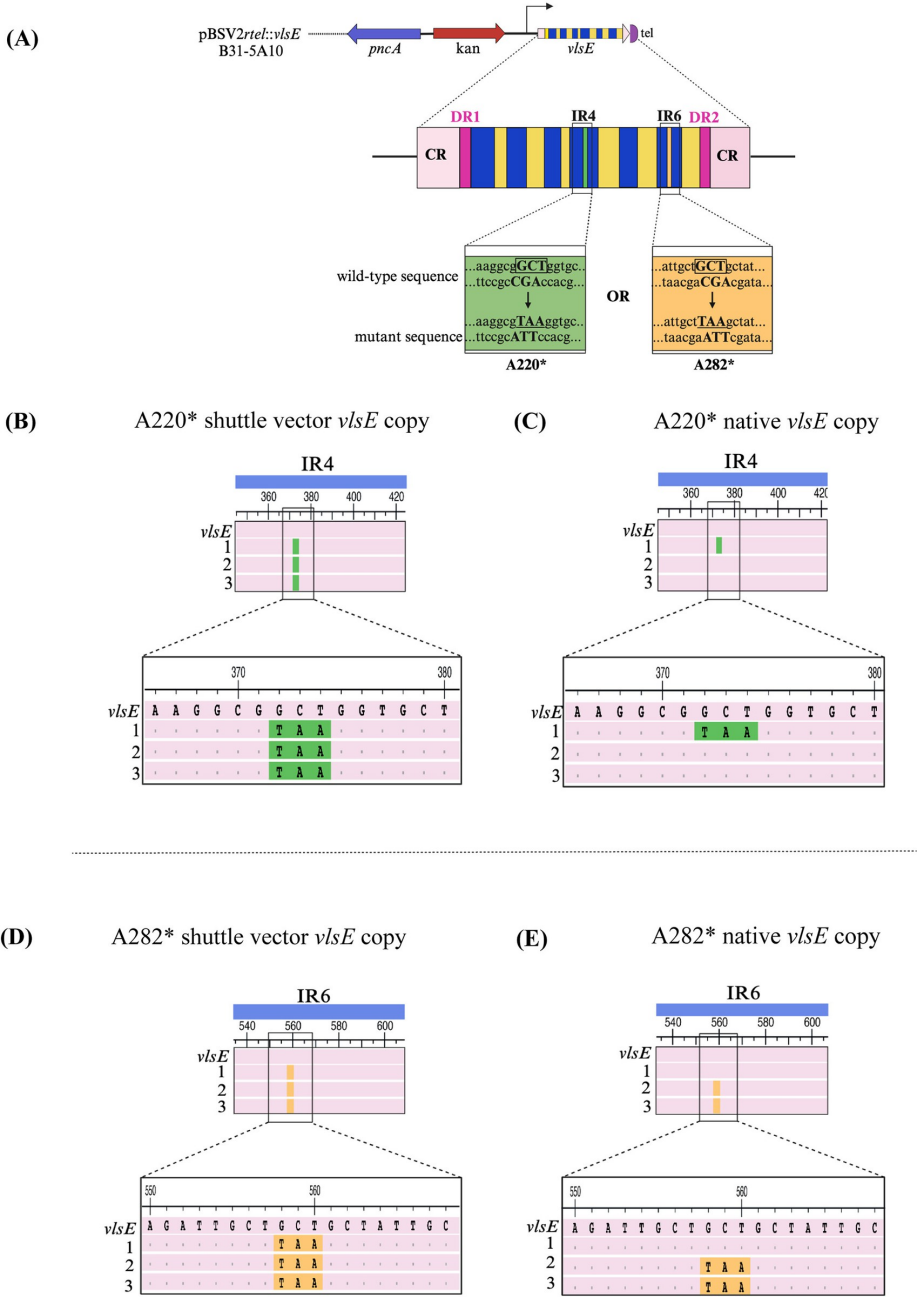
Introduction of mutations in invariable regions (IR) of the central cassette of *vlsE*. (A) A schematic representation of mutations generated in invariable regions of the central cassette of *vlsE* on the linear shuttle vector pBSV2*rtel*::*vlsE*. Alanine coded by 5’ GCT 3’ at positions 220 in IR4 (green) and 282 in IR6 (orange) in the wild type sequence (boxed) was changed to 5’ TAA 3’ coding for stop codon in the mutant sequence (underlined). CR, constant regions (pink); IR, invariable region (blue); DR direct repeat (magenta). (B) Multiple sequence alignment of *vlsE* copy on SV in A220*. PCR products from the mutant group were TOPO cloned and sequenced using M13 primers as before. All three clones retained the stop codon sequence in IR4 (green). (C) Multiple sequence alignment of the native *vlsE* copy on lp28-1 in A220*. One out of three clones sequenced for native *vlsE* in the A220* mutant group incorporated the stop codon (green). (D) Multiple sequence alignment of *vlsE* copy on SV in A282*. All three clones retained stop codon sequence in IR6 (orange). (E) Multiple sequence alignment of native *vlsE* copy on lp28-1 in A282*. Sequencing of *vlsE* amplified from native lp28-1 from IR6 mutant group showed incorporation of stop codons from SV in two clones. Color coding is same as described in Fig 5. Subpart (A) was created with BioRender.com.

After discovering the method to incorporate DR mutations into the native *vlsE* gene, which were located outside the variable cassette region, we next assessed if mutations could be introduced in other regions of the *vlsE* gene using our newly discovered method. For this, we shifted our focus towards exploring the feasibility of introducing mutations within the central cassette region. To validate this hypothesis, two constructs were generated that harbored a stop codon within one of the invariable regions of the *vlsE* central cassette. Alanine at positions 220 and 282 was independently substituted to generate the stop codon ‘TAA’. The positions of these amino acids correspond to invariable region 4 (IR4) and invariable region 6 (IR6), respectively (Fig 7A). These positions were chosen based on a previous study showing that full length VlsE protein expression is not required for murine infection and dissemination by *B*. *burgdorferi* [39]. Following repeated unsuccessful attempts to clone the synthesized genes into the pBSV2*rtel* shuttle vector, a direct transformation technique was conducted by introducing the ligation product of the insert and vector into 5A10 strain of *B*. *burgdorferi* without using transformation of *E*. *coli* as an intermediate step. Upon confirmation of the sequence and plasmid profile of the clones, groups consisting of three C3H/HeN mice each were infected subcutaneously, and progress of the infection was monitored by culturing tissues every week, following the previously outlined procedure. Table 4 summarizes the culture data showing that all the mice of both groups were infected by four weeks.

**Table 4:** Culture data showing the persistence of infection in C3H/HeN mice harboring stop codons in *vlsE* copy on shuttle plasmid in B31-5A10 strain.

B31 clone	Wk2 ear	Wk3 ear	Wk4 skin	Wk4 bladder	Wk4 heart	Wk4 joint
A220*	1/3^a^	1/3	3/3	3/3	3/3	3/3
A282*	3/3	3/3	3/3	3/3	3/3	3/3

^a^Values listed correspond to number of culture positive/number of mice tested.

Sequence analysis from the mice infected with B31-5A10/pBSV2*rtel*::*vlsE*:A220* showed the *vlsE* gene copy on the shuttle vector retained the stop codon at position 220 in all the three clones sequenced (Fig 7B). Simultaneously, the stop sequence was incorporated into the same spot of the central region of the native *vlsE* copy in one out of three clones sequenced (Fig 7C). Similarly, in B31-5A10/pBSV2*rtel*::*vlsE*:A282* with the stop codon in IR6, sequencing of all three clones showed the stop codon in the shuttle vector (Fig 7D) while two sequences manifested the integration of a stop codon into the native *vlsE* copy (Fig 7E). Thus, we showed that stop codons present on the shuttle vector within the central variable cassette of *vlsE* can also ‘move’ or recombine to the native *vlsE*. Therefore, these results once again validate the successful establishment of the system designed for introducing mutations within the native *vls* locus. This reiteration emphasizes the efficacy and reproducibility of the developed approach, solidifying its utility for targeted genetic modifications in the designated *vls* genomic locus.

## Discussion

Antigenic variation is one of the key strategies employed by *B*. *burgdorferi* to circumvent the mammalian adaptive immune system and sustain a persistent infection in the host. This process is characterized by the continuous alteration of the outer surface protein, VlsE, which renders it unrecognizable by the host’s humoral immune response [34–36, 50, 52]. Despite substantial advancements in understanding the molecular biology of this spirochete and sophisticated techniques such as next generation DNA sequencing, attempts to genetically manipulate specific components within the native *vls* locus on lp28-1 have been largely unsuccessful until now. Our study introduces the discovery of a method that, for the first time, allows manipulation of several genetic determinants within the native *vls* locus, overcoming a challenge that has persisted for more than two decades.

Our present study has led to several novel insights regarding the *vls* locus. First, we have demonstrated that gene conversion can occur *in trans* only when *vlsE* is located on a linear plasmid, underscoring the importance of plasmid topology for *in trans* gene conversion process. Second, our results indicate that the 17 bp direct repeats play an important role in *vlsE* recombination *in trans*. Third, we successfully incorporated DR mutations into *vlsE* on the native lp28-1 from a shuttle vector gene copy, demonstrating the feasibility of precise genetic manipulation within *vlsE*. This ability to introduce targeted mutations will no doubt allow for more detailed functional analyses of the genetic elements within *vlsE*. We also successfully showed the introduction of mutations in two different invariable regions (IR) within the native *vlsE*, highlighting the robustness and efficacy of our approach. Thus, our method allows for the generation of mutations both outside and within the central variable cassette region of *vlsE*. This versatility in creating targeted mutations provides a powerful tool for dissecting the functional roles of various conserved and variable regions within the *vlsE* gene.

A longstanding question in the field is whether plasmid topology influences *vlsE* recombination in *B*. *burgdorferi*. A previous study by Lawrenz et al. [52] suggested a potential *cis* requirement for *vls* recombination, implying that both *vlsE* and silent cassettes need to reside on the same plasmid. However, the shuttle vector carrying *vlsE* in that study was in a circular form, whereas the native *vls* locus is located on the linear lp28-1 plasmid. It is likely that the inability of the *vlsE* gene on the circular shuttle plasmid to undergo sequence variation led to continuous expression of the parental form of VlsE on the spirochete surface. This parental VlsE was readily recognized and targeted by host antibodies, facilitating the ability of the host immune system to clear the infection. Conversely, a *vlsE* gene copy residing on the linear shuttle plasmid allowed for sequence variation of *vlsE*, contributing to a more persistent infection. Hence, our results demonstrate that the topology of the DNA molecule carrying *vlsE* is crucial for *in trans* gene conversion. In contrast, experiments with the mini-*vls* system suggested that DNA topology does not play a significant role in *vls* gene switching [56]. That system, however, relied on gene conversion in *cis*, which may account for the difference in findings between the two studies.

As mentioned above, one possible limitation of this study is the presence of mutations in the promoter of *vlsE* gene in shuttle vector copies. While it is unclear whether the moderated expression of the *vlsE* gene copies affected the outcomes of our study, we consider this unlikely because the wild type *vlsE* and DR mutant copies exhibited markedly different levels of gene conversion despite containing the same promoter mutations. Therefore, the presence of the promoter mutations in all *B*. *burgdorferi* clones to dampen overall VlsE expression allowed for an accurate comparative analysis.

The 17 bp direct repeats flanking the central variable cassette in *vlsE* [34] are also found between most of the silent cassettes; with five cassettes exhibiting non-identical DRs, and cassettes 2 and 16 characterized by a single DR [69]. Our discovery that *vlsE* recombination can occur *in trans* opened new avenues for examining role of these 17 bp DRs. Interestingly, our data showed that these DR mutations inhibit recombination of *vlsE in trans*. This inhibition could be attributed to the critical role of sequence homology in gene conversion. A previous study has shown that silent cassettes with DRs identical to those flanking the variable region of *vlsE* have the highest frequency of being used as templates for gene conversion, whereas cassettes with the least similarity were used less frequently [69]. Consequently, when DRs on silent cassettes are no longer identical to those flanking the *vlsE* central region on the shuttle vector copy, recombination *in trans* was not observed. This suggests that these repeats may play a crucial role in the homologous recombination events that facilitate the antigenic variation of the VlsE protein. These DRs were initially proposed as crucial DNA sites facilitating recombinational switching at the *vlsE* locus [34]. However, subsequent studies revealed that these DRs are poorly conserved across different *Borrelia* species and even among various strains of *B*. *burgdorferi* [70]. This lack of conservation undermines the hypothesis that DRs have a general role in facilitating recombinational switching at the *vlsE* locus. However, based on NGS data it has been suggested that 17 bp DRs have an important role in the switching process in *B*. *burgdorferi* [70]. Nonetheless, the precise function of these DRs in recombination process remains unclear and warrants additional research, particularly in clones harboring DR mutations in the native *vlsE* gene.

The most intriguing finding of our study was the successful introduction of mutations into the native *vlsE* gene of lp28-1. As previously mentioned, the complexities associated with manipulating the native *vls* locus have substantially hindered genetic research in this area. Our study demonstrates the successful incorporation of DR mutations and stop codons into different regions of the native *vlsE* gene, both outside and within the central variable cassette region, respectively. This advancement provides a valuable tool for exploring the genetic mechanisms underlying antigenic variation. Prior to this work, there was no method available for directly introducing mutations into the *vlsE* gene at its native location, making this finding noteworthy. Research in this field has been restricted to two primary studies: one where the entire *vls* locus was deleted *in vivo* by inserting a replicated telomere (*rtel*), which was then processed by endogenous telomere resolvase, ResT, resulting in the loss of entire *vls* locus [50, 56]; and another that developed a mini-*vls* system [56]. This mini-*vls* system, which includes only *vlsE*, the intergenic region, and silent cassette 2 on lp5 based shuttle vector, does not accurately replicate the wild-type context and exhibits a markedly reduced switching frequency due to the absence of additional elements present in the full-length *vls* locus. Therefore, our method for generating mutations in the native *vlsE* gene overcomes this shortcoming and offers a plethora of opportunities to study the highly conserved genetic elements located within the native *vls* locus. This accomplishment not only advances the technical capabilities within the field but also deepens our understanding of genetic variability in the *vlsE* gene. It addresses a critical gap in spirochete biology and enables a range of possibilities for future investigations into the role of the *vlsE* gene in host-pathogen interactions, as well as the broader dynamics of bacterial persistence and adaptation. Another notable observation was that the proportion of clones with the DR2 mutation was higher compared to those with the DR1 mutation. Only one clone out of 15 showed the incorporation of the DR1 mutation. This suggests that DR1 may play a more crucial role during gene conversion of *vlsE*. This observation necessitates further investigation to elucidate the precise role of DR1 in the gene conversion process. A limitation of the current study is the absence of definitive evidence that silent *vls* cassettes recombine directly into the *vlsE* locus on the *trans* plasmid. Given this, it remains plausible that recombination occurs initially at the native *vlsE* locus, with subsequent transfer of genetic material to the trans *vlsE*. To address this possibility and refine our understanding of *vlsE* recombination, a critical next step would involve deletion of the native *vlsE* gene from lp28-1, allowing observation of recombination events exclusively at the *trans vlsE* locus. Confirmation of recombination under these conditions would provide a unique opportunity to construct variant *vlsE* loci, enabling precise mapping of essential DNA sequences and regulatory elements driving this recombination process.

The implications of this study are extensive, addressing numerous unresolved questions about the *vls* locus that continue to intrigue researchers. Dissecting the mechanism of gene conversion may help to explain the unstable nature of the *vls* locus when carried on circular plasmid. It may be that imposing this selective pressure for *vls* DNA linearity is one reason for the highly linear existence of Lyme disease *Borrelia* genomes. Additionally, our findings that *vlsE* gene conversion can occur *in trans* raise the question of whether the polyploidy nature of the *B*. *burgdorferi* genome significantly impacts VlsE antigenic variation.

In conclusion, our study represents a significant advancement in the field of spirochete biology by providing the first technique for the genetic manipulation of the native *vls* locus. It overcomes previous limitations and constitutes a preliminary framework for genetic analyses that have remained dormant for over two decades. Future research leveraging this genetic manipulation technique is expected to yield substantial insights into the pathogenesis, immune evasion, and persistence strategies of *B*. *burgdorferi*, ultimately contributing to improved prevention and treatment of Lyme disease.

## Materials and Methods

### Ethics statement

The experiments on mice were carried out according to the protocols and guidelines approved by American Association for Accreditation of Laboratory Animal Care (AAALAC) and by the Office of the Campus Veterinarian at Washington State University (Animal Welfare Assurance A3485-01 and USDA registration number 91-R-0002). These guidelines follow the U.S. Public Health Service Policy on Humane Care and Use of Laboratory Animals. The animals were housed and maintained in an AAALAC- accredited facility at Washington State University, Pullman, WA. The Washington State University Institutional Animal Care and Use Committee approved the experimental procedures carried out during the current studies.

### Bacterial strains

Detailed descriptions of *B*. *burgdorferi* strains used in these studies are presented in S2 Table. All *B*. *burgdorferi* clones were cultivated in liquid BSK-II medium supplemented with 6% rabbit serum (Cedarlane Laboratories, Burlington, NC) and incubated at 35°C under 1.5% CO_2_. Mutant strains were grown in media supplemented with kanamycin (200 μg/ml) and/or gentamicin (100 μg/ml). Cell densities and growth phase were monitored by dark-field microscopy and enumerated using a Petroff-Hausser counting chamber.

### Plasmid construction

Primers and probes used in the study are listed in S1 Table. The *vlsE* gene, along with its native promoter, was synthesized commercially by GenScript and cloned at KpnI and XbaI sites (NEB) into pBSV2 shuttle vector. *pncA* gene with its native promoter was cloned at NcoI and FseI sites (NEB). To make this circular plasmid in linear form, replicated telomere (*rtel*) was added downstream of the *vlsE* gene resulting in the pBSV2*rtel*::*vlsE* plasmid. Cloning of *rtel* has been described previously in great detail [50, 61]. Both the inserts and vector were ligated overnight at 16°C using T4 DNA ligase (Thermo Scientific). Ligated DNA was precipitated and electroporated directly into B31-5A10 cells.

Mutations in DR1 and DR2 regions were carried out by inverse PCR. Primers are listed in S1 Table, and the template pMBL20 [52] was used to convert the four guanine nucleotides to TATA. Subsequently, *pncA* along with its promoter was added at NcoI and FseI restriction sites. The *vlsE* gene was sequenced to confirm the DR mutations. Positive clones were grown in 200 ml media containing kanamycin, and DNA was extracted using Maxiprep kit (Qiagen) for transformation into *B*. *burgdorferi* cells.

The *vlsE* gene, along with its native promoter, harboring stop codons at positions 220 (IR4) and 282 (IR6) which substituted the respective alanine residues, was synthesized commercially by GenScript. The synthesized genes were cloned in plasmids pCC1 and were transformed into EPI300 competent cells. The clones were grown in 100 ml LB media supplemented with appropriate antibiotics and induced for minimum of four hours by induction solution provided by GenScript. DNA was isolated using Midiprep kit (Qiagen). Both the inserts and vector (pBSV2*rtel*::*vlsE*) were digested by KpnI and XbaI restriction endonucleases (NEB) and extracted from the respective gels. The fragments were ligated at a 3:1 insert-to-vector ratio in a total volume of 20 μl, using T4 DNA ligase (Thermo Scientific). The reaction was carried out overnight at 16°C in 6–8 tubes. The ligated DNA was pooled together, precipitated using phenol-chloroform method, and electroporated directly into B31-5A10 cells.

### *B*. *burgdorferi* transformation

*Borrelia burgdorferi* B31-5A10 or A1 cells were electroporated as described previously [50]. DNA from culture-positive wells was extracted using the phenol-chloroform method and used for PCR analysis to confirm the presence of shuttle vector. Plasmid content for each verified transformant was determined by PCR using plasmid-specific primers as previously described [71].

### RNA extraction and gene expression analysis by qRT-PCR

Three 5 ml cultures of each *Borrelia* strain were grown to late log phase (8 x10^7^ to 1 x 10^8^ cells/ml), and RNA was isolated using the RNeasy Mini Kit (Qiagen). DNA contamination was removed using the TURBO DNA-free kit (Invitrogen), per manufacturer’s instructions. cDNA for all samples was synthesized using the iScript cDNA Synthesis kit (Bio-Rad Laboratories). Following generation of cDNA, droplet digital PCR was performed using the QX200 Droplet Digital PCR (ddPCR) system (Bio-Rad Laboratories) as carried out before [72]. The *vlsE* and *flaB* genes were amplified using primers and TaqMan probes shown in S1 Table. Raw data to generate graph in Fig 3B is provided in S1 GraphData.

### Proteinase K accessibility assay and Immunoblotting

The proteinase K digestion assay was performed as described previously [51]. Briefly, 10^8^ spirochetes were incubated in phosphate-buffered saline (PBS) in the absence or presence of proteinase K (Sigma) at a concentration of 200 μg/mL for 20 minutes, treated with phenylmethylsulfonyl fluoride (Sigma), centrifuged, and resuspended in PBS for subsequent immunoblot analysis using antibodies against VlsE or FlaB.

### Field inversion gel electrophoresis and Southern blot hybridization

*B*. *burgdorferi* total DNA was isolated by phenol-chloroform extraction, followed by isopropanol precipitation. Multiple lanes containing 1.5 μg of DNA per lane were separated on 0.65% agarose gel electrophoresis. The gel was run initially at 65 V for 45 minutes before applying the periodic inversion of the electric field direction for additional 16–18 h. To keep the temperature of the electrophoresis gel in control, buffer was recirculated continuously using a pump over the course of the experiment. Bands containing native lp28-1 (28 kb) and shuttle vector plasmid (8.6 kb) were excised from the gels separately. The shuttle vector was extracted utilizing the QIAquick Gel Extraction Kit (Qiagen), whereas the 28 kb plasmid was isolated using the QIAEX II Gel Extraction Kit (Qiagen). Purified DNA was then used as a template for subsequent PCR.

Southern blot hybridization was done as described before [55]. Probes for *vlsE* and kanamycin were generated from plasmid template DNA using primers indicated in S1 Table with the DIG Probe Synthesis kit (Roche), per manufacturer’s instructions. Bands were detected with the DIG Luminescent Detection kit (Roche).

### Mouse infections

Four- to six-week-old immunocompetent C3H/HeN mice (C3H; Jackson, Sacramento, CA) were infected by subcutaneous needle inoculation with 10^5^ total spirochetes. Three mice per group were used. The presence or absence of spirochetes was determined from blood samples (50 μl via saphenous vein) collected one week post infection, or from tissue samples (ear, heart, joint and bladder) harvested two weeks post infection. Blood and tissues were incubated in BSK-II medium supplemented with a mixture of antibiotic and antifungal drugs (20 μg/ml phosphomycin, 50 μg/ml rifampin, and 2.5 μg/ml amphotericin B) to prevent the growth of contaminating bacteria and fungi. The cultures were examined by dark field microscopy for the presence or absence of spirochetes following four to seven days of incubation. The tissues from individual mice were also incubated in BSK-II medium containing gentamicin or kanamycin to ensure that the recovered *B*. *burgdorferi* clones had not lost the shuttle plasmid vector.

### RFLP analysis

PCR-RFLP assay was performed as described previously [50]. Briefly, a 775 bp segment containing the six variable regions of *vlsE* was PCR amplified using the primers P243 and P244 (S1 Table). PCR products were purified using QIAquick PCR spin columns (Qiagen), quantified by absorbance at 260 nm, and digested in reactions containing 200 ng DNA and 2 units of HphI (NEB) as per the manufacturer’s instructions at 37°C for 1 h. Reactions were analyzed on a 2% agarose gel.

### DNA sequencing

*vlsE* variable region was PCR amplified using primers P243 and P244. Phusion high-fidelity DNA polymerase (Thermo Scientific) was used throughout the studies. The amplicon was cloned in pCR Blunt II-TOPO vector and transformed into one shot chemically competent *E*. *coli* Top10 cells (Invitrogen). Plasmid DNA was extracted using mini-prep kit (Qiagen). *vlsE* fragment was sequenced using M13 forward and reverse primers by Eurofins Genomics (Kentucky). The sequences were aligned using Clustal Omega algorithm in SnapGene, and the figures were created with the NCBI Multiple Sequence Alignment Viewer (MSA) 1.25.0.

## Supporting information

S1 Fig Expression and surface localization of VlsE. Viable spirochetes in strains A1, WT (pBSV2*rtel*::*vlsE*), and DR mutant (pBSV2*rtel*::*vlsE*:DR) were treated with (+ProK) or without (-ProK) proteinase K for 20 min followed by SDS-polyacrylamide gel electrophoresis (10^8^ cells/lane) and Western blotting. Two identical blots were processed with anti-VlsE or anti-FlaB antibodies. There was no difference in the VlsE expression levels between WT and DR mutant. Treatment with proteinase K dramatically reduced VlsE immunostaining for the WT and mutant clones.
(TIF)

S2 Fig VlsE expression with respect to mutations in promoter region. Shuttle vectors used in the current study contained two mutations in promoter region of *vlsE*. These mutations (Mut_P) reduced the expression of VlsE when compared to strain with wild type promoter sequence (Wt_P). Equal number of cells (10^8^ cells/lane) were separated on SDS-PAGE and immunoblotted with antibodies specific to VlsE and FlaB.
(TIF)

S3 Fig Southern blot analysis of DR mutants in pBSV2*rtel*::*vlsE* shuttle vector in 5A10 strain. Total DNA was extracted from 5A10 transformants containing the linear plasmid with DR mutations, subjected to field-inversion gel electrophoresis and subsequently probed with DIG labeled (i) *vlsE* and (ii) kanamycin probes. Distinct hybridization bands were observed at 28 kb and 8.6 kb, corresponding to native *vlsE* on lp28-1 and its copy on the shuttle vector, respectively, when the membrane was probed with *vlsE* gene sequence. Additionally, a single band at 8.6 kb was detected for the kanamycin resistance gene when probed with kanamycin-specific probe. M, DIG labelled DNA molecular weight marker II; 1–8 clones, 5A10, background strain; Ec +ve, positive control plasmid in *E*.*coli*.
(TIF)

S4 Fig DNA sequences of WT *vlsE* copy on shuttle vector. Complete DNA sequences of WT copy of *vlsE* on the linear shuttle vector used in multiple sequence alignment in Fig 5B. One representative sequence from each of the three mice were aligned with *in vitro* grown *vlsE*. M1, M2, M3 indicates mouse number. The alignment spans the positions 1–660, starting at the *vlsE* sequence where primer P243 binds the N-terminal constant region and ending at 16 bp of the C-terminal constant region.
(PDF)

S5 Fig DNA sequences of *vlsE* copy on shuttle vector in DR mutant group. Complete DNA sequences of DR mutant copy of *vlsE* on the linear shuttle vector used in multiple sequence alignment in Fig 5C. 15 clones were randomly selected after limiting dilution of the spirochetes recovered from the bladder tissue from each mouse and sequenced as above. The alignment spans the positions 1–660, starting at the *vlsE* sequence where primer P243 binds the N-terminal constant region and ending at 16 bp of the C-terminal constant region.
(PDF)

S6 Fig DNA sequences of *vlsE* copy on native lp28-1 in DR mutant group. Complete DNA sequences of native *vlsE* on lp28-1 used in multiple sequence alignment in Fig 6B in DR mutant group. The alignment spans the positions 1–660, starting at the *vlsE* sequence where primer P243 binds the N-terminal constant region and ending at 16 bp of the C-terminal constant region.
(PDF)

S7 Fig Plasmid incompatibility. Southern blot analysis 5A10 transformants with single copy of *vlsE* on native lp28-1. The pBSV2G::*pncA* plasmid was transformed into mutants containing the DR2 mutation in native *vlsE* to eliminate pBSV2*rtel*::*vlsE*. Total DNA was extracted from transformants, subjected to field-inversion gel electrophoresis and subsequently probed with DIG labeled (i) kanamycin and (ii) *vlsE* probes. All clones had successfully eliminated the original shuttle vector and had only a single copy of *vlsE* on native lp28-1. M, DIG labelled DNA molecular weight marker II; 1–6 clones; +ve, positive control- 5A10 clone having both native lp28-1 and shuttle vector *vlsE* copies.
(TIF)

S1 Table Oligos used in the study. (DOCX)

S2 Table Strains and plasmids used in the study. (DOCX)

S1 GraphData Raw data to generate graph in Fig 3B is provided. (XLSX)
