# Supplementary figures and images for: Breaking a barrier: In trans vlsE recombination and genetic manipulation of the native vlsE gene of the Lyme disease pathogen
Source: PLoS Pathog. 2025 Jan 10;21(1):e1012871. doi: 10.1371/journal.ppat.1012871 (PMC11756760; doi:10.1371/journal.ppat.1012871)

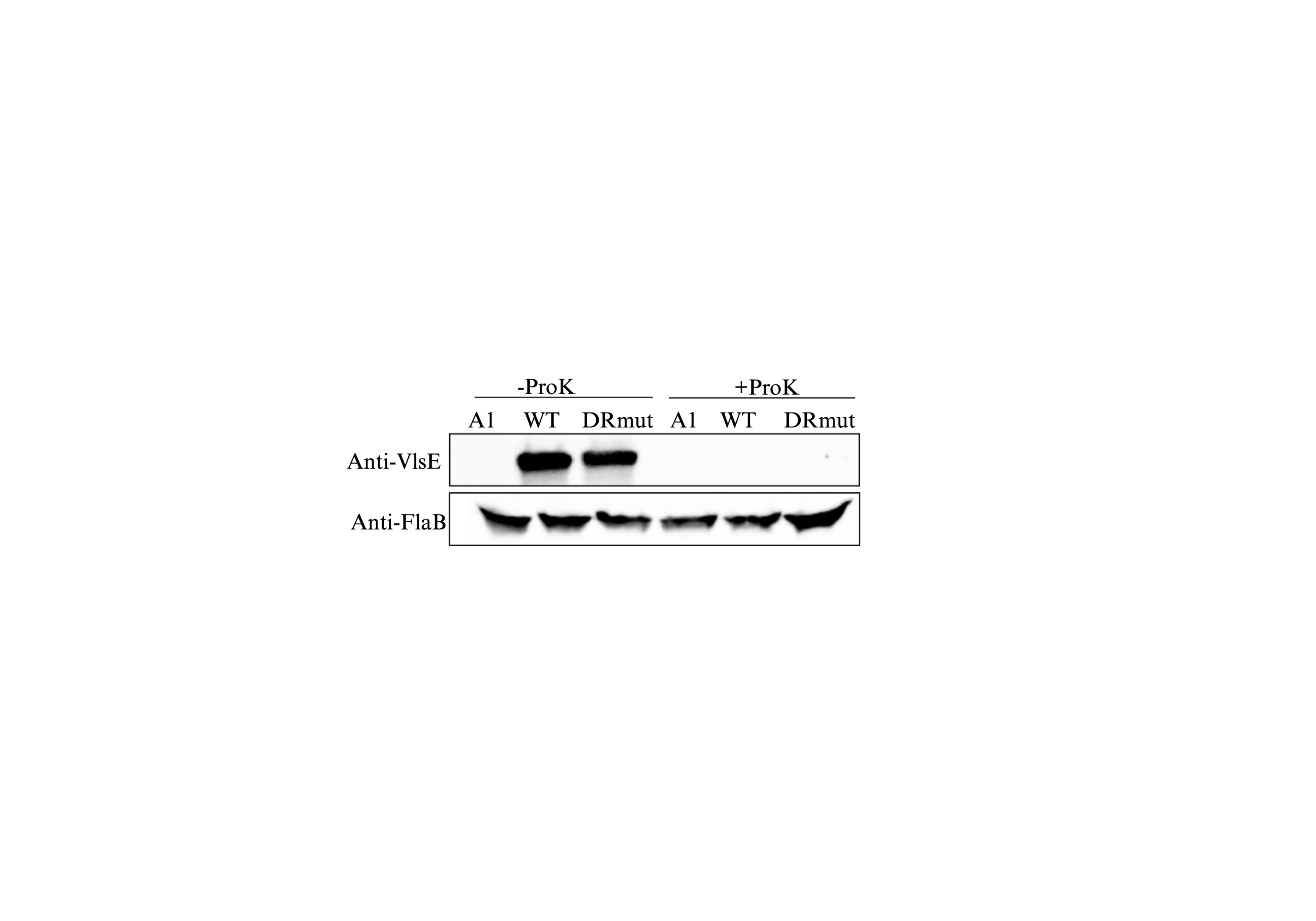

Supplement: S1 Fig — Viable spirochetes in strains A1, WT (pBSV2rtel::vlsE), and DR mutant (pBSV2rtel::vlsE:DR) were treated with (+ProK) or without (-ProK) proteinase K for 20 min followed by SDS-polyacrylamide gel electrophoresis (108 cells/lane) and Western blotting. Two identical blots were processed with anti-VlsE or anti-FlaB antibodies. There was no difference in the VlsE expression levels between WT and DR mutant. Treatment with proteinase K dramatically reduced VlsE immunostaining for the WT and mutant clones. (TIF) [file ppat.1012871.s001.tif]

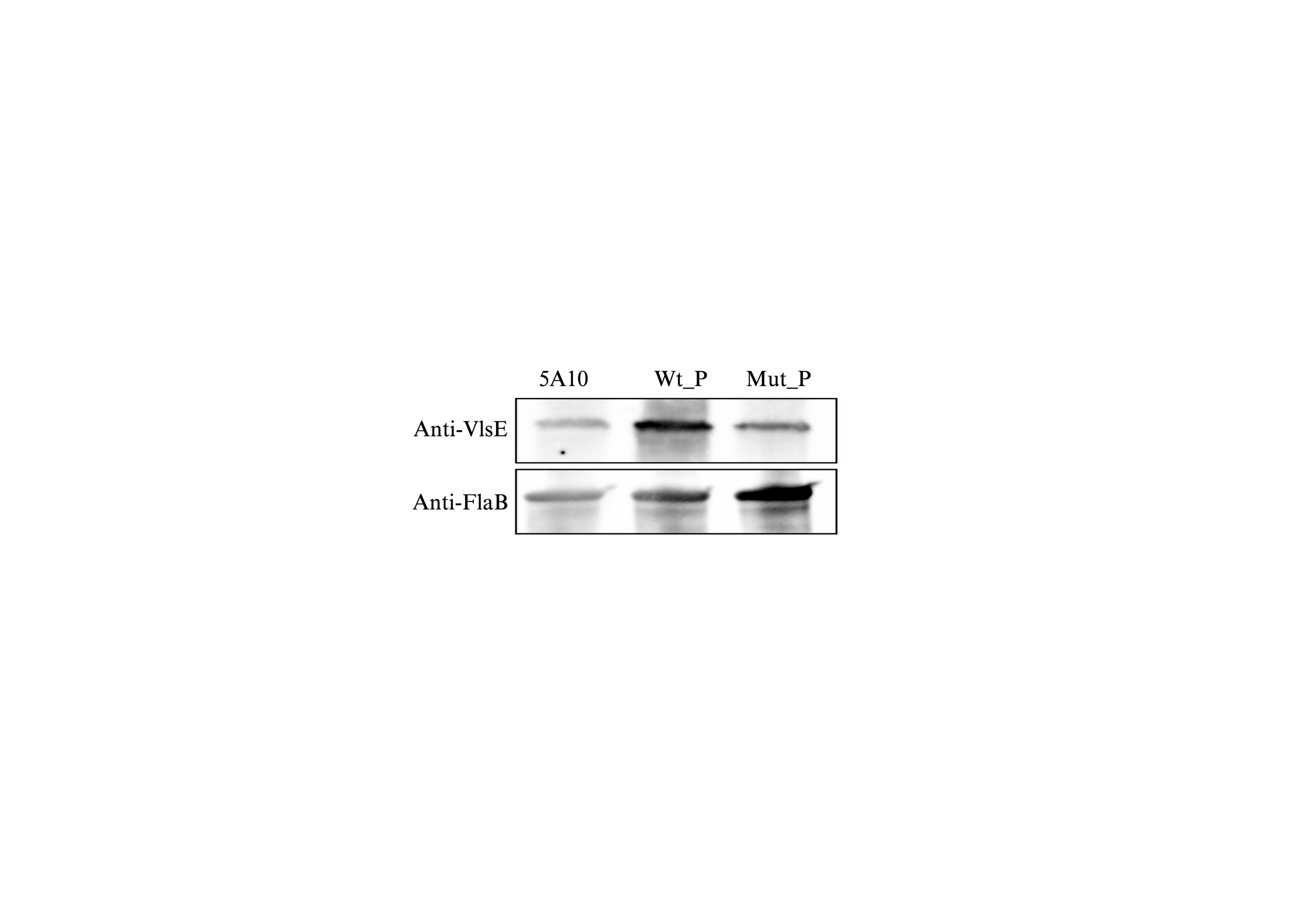

Supplement: S2 Fig — Shuttle vectors used in the current study contained two mutations in promoter region of vlsE. These mutations (Mut_P) reduced the expression of VlsE when compared to strain with wild type promoter sequence (Wt_P). Equal number of cells (108 cells/lane) were separated on SDS-PAGE and immunoblotted with antibodies specific to VlsE and FlaB. (TIF) [file ppat.1012871.s002.tif]

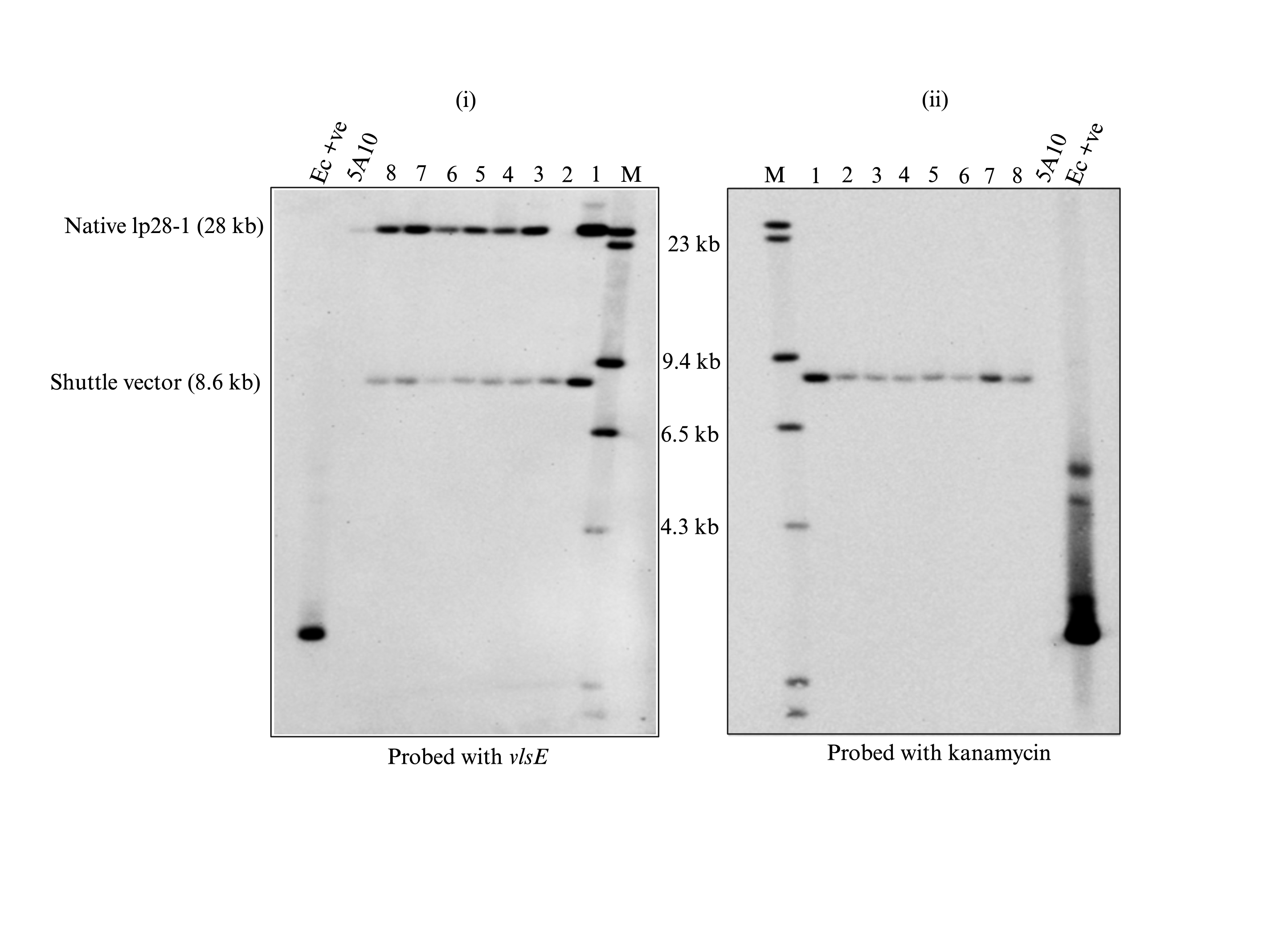

Supplement: S3 Fig — Total DNA was extracted from 5A10 transformants containing the linear plasmid with DR mutations, subjected to field-inversion gel electrophoresis and subsequently probed with DIG labeled (i) vlsE and (ii) kanamycin probes. Distinct hybridization bands were observed at 28 kb and 8.6 kb, corresponding to native vlsE on lp28-1 and its copy on the shuttle vector, respectively, when the membrane was probed with vlsE gene sequence. Additionally, a single band at 8.6 kb was detected for the kanamycin resistance gene when probed with kanamycin-specific probe. M, DIG labelled DNA molecular weight marker II; 1–8 clones, 5A10, background strain; Ec +ve, positive control plasmid in E.coli. (TIF) [file ppat.1012871.s003.tif]

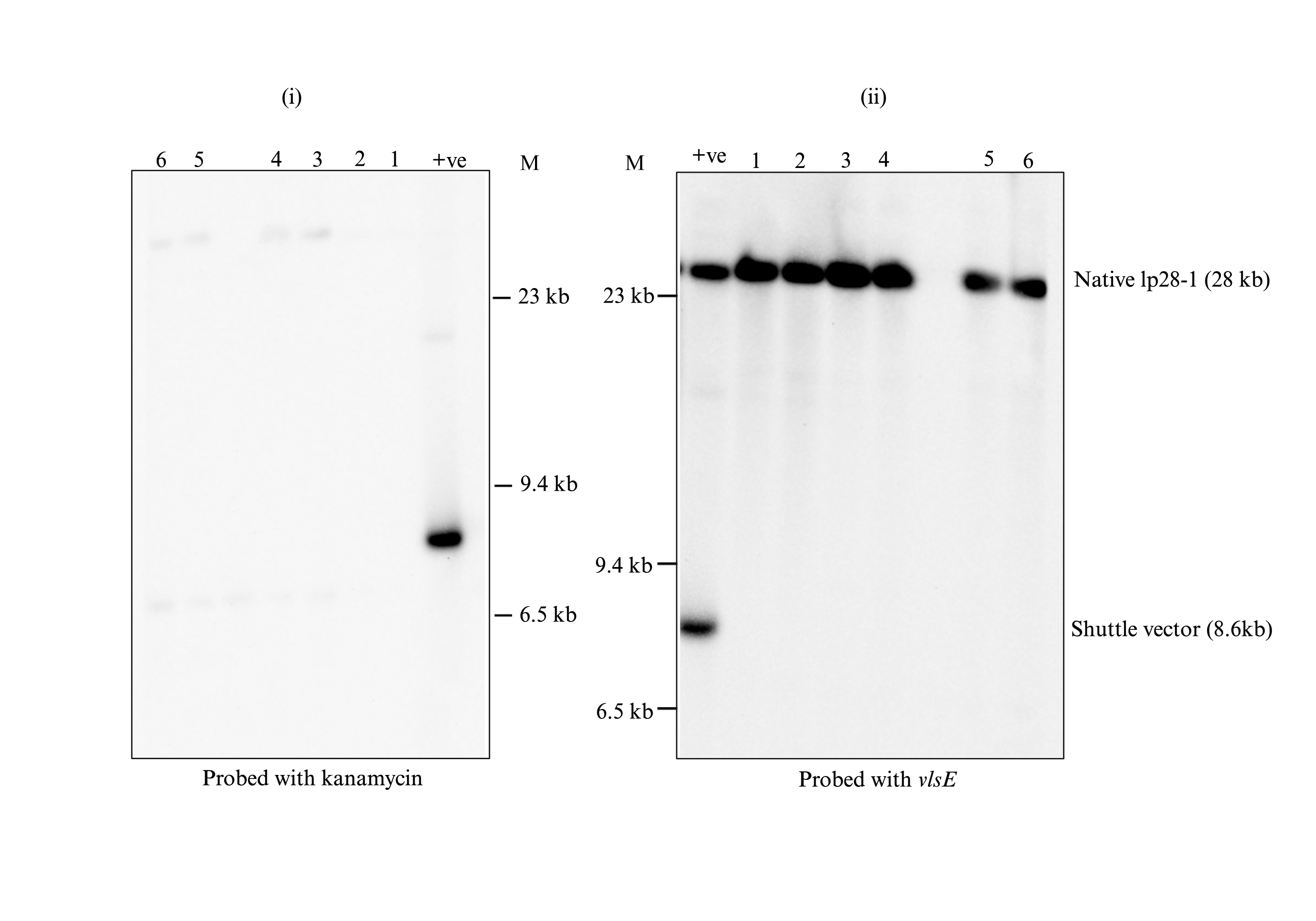

Supplement: S7 Fig — The pBSV2G::pncA plasmid was transformed into mutants containing the DR2 mutation in native vlsE to eliminate pBSV2rtel::vlsE. Total DNA was extracted from transformants, subjected to field-inversion gel electrophoresis and subsequently probed with DIG labeled (i) kanamycin and (ii) vlsE probes. All clones had successfully eliminated the original shuttle vector and had only a single copy of vlsE on native lp28-1. M, DIG labelled DNA molecular weight marker II; 1–6 clones; +ve, positive control- 5A10 clone having both native lp28-1 and shuttle vector vlsE copies. (TIF) [file ppat.1012871.s007.tif]
