# Supplementary material for: Breaking a barrier: In trans vlsE recombination and genetic manipulation of the native vlsE gene of the Lyme disease pathogen
Source: PLoS Pathog. 2025 Jan 10;21(1):e1012871. doi: 10.1371/journal.ppat.1012871 (PMC11756760; doi:10.1371/journal.ppat.1012871)
Supplement: S4 Fig — Complete DNA sequences of WT copy of vlsE on the linear shuttle vector used in multiple sequence alignment in Fig 5B. One representative sequence from each of the three mice were aligned with in vitro grown vlsE. M1, M2, M3 indicates mouse number. The alignment spans the positions 1–660, starting at the vlsE sequence where primer P243 binds the N-terminal constant region and ending at 16 bp of the C-terminal constant region. (PDF) [file ppat.1012871.s004.pdf]

```
>vlsE
GCGATATAAGTAGTACGACGGGGAAACCAGATAGTACAGGTTCTGTTGGAACTGCCGTTGAGGGGGCTAT
TAAGGAAGTT
AGCGAGTTGTTGGATAAGCTGGTAAAAGCTGTAAAGACAGCTGAGGGGGCTTCAAGTGGTACTGCTGCAA
TTGGAGAAGT
TGTGGCTGATGCTGATGCTGCAAAGGTTGCTGATAAGGCGAGTGTGAAGGGGATTGCTAAGGGGATAAAG
GAGATTGTTG
AAGCTGCTGGGGGGAGTGAAAAGCTGAAAGCTGTTGCTGCTGCTAAAGGGGAGAATAATAAAGGGGCAGG
GAAGTTGTTT
GGGAAGGCT------
GGTGCTGCTGCTCATGGGGACAGTGAGGCTGCTAGCAAGGCGGCTGGTGCTGTTAGTGCTGTTAG
TGGGGAGCAGATATTAAGTGCGATTGTTACGGCTGC------
TGATGCGGCTGAGCAGGATGGAAAGAAGCCTGAGGAGG
CTAAAAATCCGATTGCTGCTGCTATTGGGGATAAAGATGGGGGT---
GCGGAGTTTGGTCAGGATGAGATGAAGAAGGAT
GATCAGATTGCTGCTGCTATTGCTTTGAGGGGGATGGCTAAGGATGGAAAGTTTGCTGTGAAGGATGGTG
AGAAAGAGAA
GGCTGAGGGGGCTATTAAGGGAGCTGCTGAGTCTGCAGTTCGCAAAGTTTTAGG

>M1
GCGATATAAGTAGTACGACGGGGAAACCAGATAGTACAGGTTCTGTTGGAACTGCCGTTGAGGGGGCTAT
TAAGGAAGTT
AGCGAGTTGTTGGATAAGCTGGTAAAAGCTGTAAAGACAGCTGAGGGGGCTTCAAGTGGTACTGCTGCAA
TTGGAGAAGT
TGTGGATAAT------
GCTGCGAAGGCTGCTGATAAGGCGAGTGTGACGGGGATTGCTAAGGGGATAAAGGAGATTGTTG
AAGCTGCTGGGGGGAGTGAAAAGCTGAAAGTTGCTGCTGCTACAGGGG---
AGAATAATAAAGGGGCAGGGAAGTTGTTT
GGGAAGGTT------
GATGCTGCTCATGCTGGGGACAGTGAGGCTGCTAGCAAGGCGGCTGGTGCTGTTAGTGCTGTTAG
TGGGGAGCAGATATTAAGTGCGATTGTTAAGGCTGCGGCTGCTGGTGCGGCTGAGCAGGATGGAGAGAAG
CCTGGGGAGG
CTAAAAATCCGATTGCTGCTGCTATTGGGAAGGGTAATGAGAAT---
GGTGCGGAGTTTGGTGATGGGATGAAGAAGGAT
GATCAGATTGCTGCTGCTATTGCTTTGAGGGGGATGGCTAAGGATGGAAAGTTTGCTGTGAAGAAGGATG
AGAAAGGGAA
GGCTGAGGGGGCTATTAAGGGAGCTGCTGAGTCTGCAGTTCGCAAAGTTTTAGG

>M2
GCGATATAAGTAGTACGACGGGGAAACCAGATAGTACAGGTTCTGTTGGAACTGCCGTTGAGGGGGCTAT
TAAGGAAGTT
AGCGAGTTGTTGGATAAGCTGGTAAAAGCTGTAAAGACAGCTGAGGGGGCTTCAAGTGGTACTGCTGCAA
TTGGAGAAGT
TGTGGCTGAT------
GATGCTAAGGTTGCTGATAAGGAGAGTGTGACGGGGATTGCTAAGGGGATAAAGGAGATTGTTG
AAGCTGCTGGGGGGAGTGAAAAGCTGAAAGCTGTTGCTGCTGCTAAAGAGGGCAA---
TGAAAAGGCAGGGAAGTTGTTT
GGGAAGGCT------
GGTGCTGATGCTCATGGGGACAGTGAGGCTGCTAGCAAGGCGGCTGGTGCTGTTAGTGCTGTTAG
TGGGGAGCAGATATTAAGTGCGATTGTTACGGCTGC------
```

TGGTGCGGCTGAGCAGGAGGGAAAGAAGCCTGAGGAGG
CTAAAAATCCGATTGCTGCTGCTATTGGGGATAAAGATGGGGGT---GCGGAGTTTGA---
GGATGAGATGAAGAAGGAT
GATCAGATTGCTGCTGCTATTGCTTTGAGGGGGATGGCTAAGGATGGAAAGTTTGCTGTGAAGGATGGTG
AGAAAGGGAA
GGCTGAGGGGGCTATTAAGGGAGCTGCTGAGTCTGCAGTTCGCAAAGTTTTAGG

>M3
GCGATATAAGTAGTACGACGGGGAAACCAGATAGTACAGGTTCTGTTGGAACTGCCGTTGAGGGGGCTAT
TAAGGAAGTT
AGCGAGTTGTTGGATAAGCTGGTAAAAGCTGTAAAGACAGCTGAGGGGGCTTCAAGTGGTACTGCTGCAA
TTGGAGAAGT
TGTGGCTGATGATGCTGCTGCGAAGGCTGCTGATAAGGATAGTGTGACGGGGATTGCTAAGGGGATAAAG
GAGATTGTTG
AAGCTGCTGGGGGGAGTGAAAAGCTGAAAGCTGTTGCTGCTGAAGGGG---
AGAATAATAAAGAGGCAGGGAAGTTGTTT
GGGAAGGCTGGTGCTGGTGCTGGTGCTCATGGGGACAGTGAGGCTGCTAGCAAGGCGGCTGGTGCTGTTA
GTGCTGTTAG
TGGGGAGCAGATATTAAGTGCGATTGTTACGGCTGC------
TGGTGCGGCTGAGCAGGAGGGAAAGAAGCCTGCAGATG
CTACAAATCCGATTGCTGCTGCTATTGGGAAGGGTGATGCGGAGAATGGTGCGGATTTTGGTGATGGGAT
GAAGAAGGAT
GATCAGATTGCTGCTGCTATTGCTTTGAGGGGGATGGCTAAGGATGGAAAGTTTGCTGTGAAGGATGGTG
AGAAAGAGAA
GGCTGAGGGGGCTATTAAGGGAGCTGCTGAGTCTGCAGTTCGCAAAGTTTTAGG
