# Supplementary material for: Breaking a barrier: In trans vlsE recombination and genetic manipulation of the native vlsE gene of the Lyme disease pathogen
Source: PLoS Pathog. 2025 Jan 10;21(1):e1012871. doi: 10.1371/journal.ppat.1012871 (PMC11756760; doi:10.1371/journal.ppat.1012871)
Supplement: S5 Fig — Complete DNA sequences of DR mutant copy of vlsE on the linear shuttle vector used in multiple sequence alignment in Fig 5C. 15 clones were randomly selected after limiting dilution of the spirochetes recovered from the bladder tissue from each mouse and sequenced as above. The alignment spans the positions 1–660, starting at the vlsE sequence where primer P243 binds the N-terminal constant region and ending at 16 bp of the C-terminal constant region. (PDF) [file ppat.1012871.s005.pdf]

```
>vlsE
GCGATATAAGTAGTACGACGGGGAAACCAGATAGTACAGGTTCTGTTGGAACTGCCGTTGAGGGGGCTAT
TAAGGAAGTT
AGCGAGTTGTTGGATAAGCTGGTAAAAGCTGTAAAGACAGCTGAGGGGGCTTCAAGTGGTACTGCTGCAA
TTGGAGAAGT
TGTGGCTGATGCTGATGCTGCAAAGGTTGCTGATAAGGCGAGTGTGAAGGGGATTGCTAAGGGGATAAAG
GAGATTGTTG
AAGCTGCTGGGGGGAGTGAAAAGCTGAAAGCTGTTGCTGCTGCTAAAGGGGAGAATAATAAAGGGGCAGG
GAAGTTGTTT
GGGAAGGCTGGTGCTGCTGCTCATGGGGACAGTGAGGCTGCTAGCAAGGCGGCTGGTGCTGTTAGTGCTG
TTAGTGGGGA
GCAGATATTAAGTGCGATTGTTACGGCTGCTGATGCGGCTGAGCAGGATGGAAAGAAGCCTGAGGAGGCT
AAAAATCCGA
TTGCTGCTGCTATTGGGGATAAAGATGGGGGTGCGGAGTTTGGTCAGGATGAGATGAAGAAGGATGATCA
GATTGCTGCT
GCTATTGCTTTGAGGGGGATGGCTAAGGATGGAAAGTTTGCTGTGAAGGATGGTGAGAAAGAGAAGGCTG
AGGGGGCTAT
TAAGGGAGCTGCTGAGTCTGCAGTTCGCAAAGTTTTAGG

>1
GCGATATAAGTAGTACGACGGGGAAACCAGATAGTACAGGTTCTGTTGGAACTGCCGTTGAGTATACTAT
TAAGGAAGTT
AGCGAGTTGTTGGATAAGCTGGTAAAAGCTGTAAAGACAGCTGAGGGGGCTTCAAGTGGTACTGCTGCAA
TTGGAGAAGT
TGTGGCTGATGCTGATGCTGCAAAGGTTGCTGATAAGGCGAGTGTGAAGGGGATTGCTAAGGGGATAAAG
GAGATTGTTG
AAGCTGCTGGGGGGAGTGAAAAGCTGAAAGCTGTTGCTGCTGCTAAAGGGGAGAATAATAAAGGGGCAGG
GAAGTTGTTT
GGGAAGGCTGGTGCTGCTGCTCATGGGGACAGTGAGGCTGCTAGCAAGGCGGCTGGTGCTGTTAGTGCTG
TTAGTGGGGA
GCAGATATTAAGTGCGATTGTTACGGCTGCTGATGCGGCTGAGCAGGATGGAAAGAAGCCTGAGGAGGCT
AAAAATCCGA
TTGCTGCTGCTATTGGGGATAAAGATGGGGGTGCGGAGTTTGGTCAGGATGAGATGAAGAAGGATGATCA
GATTGCTGCT
GCTATTGCTTTGAGGGGGATGGCTAAGGATGGAAAGTTTGCTGTGAAGGATGGTGAGAAAGAGAAGGCTG
AGTATACTAT
TAAGGGAGCTGCTGAGTCTGCAGTTCGCAAAGTTTTAGG

>2
GCGATATAAGTAGTACGACGGGGAAACCAGATAGTACAGGTTCTGTTGGAACTGCCGTTGAGTATACTAT
TAAGGAAGTT
AGCGAGTTGTTGGATAAGCTGGTAAAAGCTGTAAAGACAGCTGAGGGGGCTTCAAGTGGTACTGCTGCAA
TTGGAGAAGT
TGTGGCTGATGCTGATGCTGCAAAGGTTGCTGATAAGGCGAGTGTGAAGGGGATTGCTAAGGGGATAAAG
GAGATTGTTG
AAGCTGCTGGGGGGAGTGAAAAGCTGAAAGCTGTTGCTGCTGCTAAAGGGGAGAATAATAAAGGGGCAGG
GAAGTTGTTT
GGGAAGGCTGGTGCTGCTGCTCATGGGGACAGTGAGGCTGCTAGCAAGGCGGCTGGTGCTGTTAGTGCTG
TTAGTGGGGA
GCAGATATTAAGTGCGATTGTTACGGCTGCTGATGCGGCTGAGCAGGATGGAAAGAAGCCTGAGGAGGCT
```

AAAAATCCGA
TTGCTGCTGCTATTGGGGATAAAGATGGGGGTGCGGAGTTTGGTCAGGATGAGATGAAGAAGGATGATCA
GATTGCTGCT
GCTATTGCTTTGAGGGGGATGGCTAAGGATGGAAAGTTTGCTGTGAAGGATGGTGAGAAAGAGAAGGCTG
AGTATACTAT
TAAGGGAGCTGCTGAGTCTGCAGTTCGCAAAGTTTTAGG

>3
GCGATATAAGTAGTACGACGGGGAAACCAGATAGTACAGGTTCTGTTGGAACTGCCGTTGAGTATACTAT
TAAGGAAGTT
AGCGAGTTGTTGGATAAGCTGGTAAAAGCTGTAAAGACAGCTGAGGGGGCTTCAAGTGGTACTGCTGCAA
TTGGAGAAGT
TGTGGCTGATGCTGATGCTGCAAAGGTTGCTGATAAGGCGAGTGTGAAGGGGATTGCTAAGGGGATAAAG
GAGATTGTTG
AAGCTGCTGGGGGGAGTGAAAAGCTGAAAGCTGTTGCTGCTGCTAAAGGGGAGAATAATAAAGGGGCAGG
GAAGTTGTTT
GGGAAGGCTGGTGCTGCTGCTCATGGGGACAGTGAGGCTGCTAGCAAGGCGGCTGGTGCTGTTAGTGCTG
TTAGTGGGGA
GCAGATATTAAGTGCGATTGTTACGGCTGCTGATGCGGCTGAGCAGGATGGAAAGAAGCCTGAGGAGGCT
AAAAATCCGA
TTGCTGCTGCTATTGGGGATAAAGATGGGGGTGCGGAGTTTGGTCAGGATGAGATGAAGAAGGATGATCA
GATTGCTGCT
GCTATTGCTTTGAGGGGGATGGCTAAGGATGGAAAGTTTGCTGTGAAGGATGGTGAGAAAGAGAAGGCTG
AGTATACTAT
TAAGGGAGCTGCTGAGTCTGCAGTTCGCAAAGTTTTAGG

>4
GCGATATAAGTAGTACGACGGGGAAACCAGATAGTACAGGTTCTGTTGGAACTGCCGTTGAGTATACTAT
TAAGGAAGTT
AGCGAGTTGTTGGATAAGCTGGTAAAAGCTGTAAAGACAGCTGAGGGGGCTTCAAGTGGTACTGCTGCAA
TTGGAGAAGT
TGTGGCTGATGCTGATGCTGCAAAGGTTGCTGATAAGGCGAGTGTGAAGGGGATTGCTAAGGGGATAAAG
GAGATTGTTG
AAGCTGCTGGGGGGAGTGAAAAGCTGAAAGCTGTTGCTGCTGCTAAAGGGGAGAATAATAAAGGGGCAGG
GAAGTTGTTT
GGGAAGGCTGGTGCTGCTGCTCATGGGGACAGTGAGGCTGCTAGCAAGGCGGCTGGTGCTGTTAGTGCTG
TTAGTGGGGA
GCAGATATTAAGTGCGATTGTTACGGCTGCTGATGCGGCTGAGCAGGATGGAAAGAAGCCTGAGGAGGCT
AAAAATCCGA
TTGCTGCTGCTATTGGGGATAAAGATGGGGGTGCGGAGTTTGGTCAGGATGAGATGAAGAAGGATGATCA
GATTGCTGCT
GCTATTGCTTTGAGGGGGATGGCTAAGGATGGAAAGTTTGCTGTGAAGGATGGTGAGAAAGAGAAGGCTG
AGTATACTAT
TAAGGGAGCTGCTGAGTCTGCAGTTCGCAAAGTTTTAGG

>5
GCGATATAAGTAGTACGACGGGGAAACCAGATAGTACAGGTTCTGTTGGAACTGCCGTTGAGTATACTAT
TAAGGAAGTT
AGCGAGTTGTTGGATAAGCTGGTAAAAGCTGTAAAGACAGCTGAGGGGGCTTCAAGTGGTACTGCTGCAA
TTGGAGAAGT

TGTGGCTGATGCTGATGCTGCAAAGGTTGCTGATAAGGCGAGTGTGAAGGGGATTGCTAAGGGGATAAAG
GAGATTGTTG
AAGCTGCTGGGGGGAGTGAAAAGCTGAAAGCTGTTGCTGCTGCTAAAGGGGAGAATAATAAAGGGGCAGG
GAAGTTGTTT
GGGAAGGCTGGTGCTGCTGCTCATGGGGACAGTGAGGCTGCTAGCAAGGCGGCTGGTGCTGTTAGTGCTG
TTAGTGGGGA
GCAGATATTAAGTGCGATTGTTACGGCTGCTGATGCGGCTGAGCAGGATGGAAAGAAGCCTGAGGAGGCT
AAAAATCCGA
TTGCTGCTGCTATTGGGGATAAAGATGGGGGTGCGGAGTTTGGTCAGGATGAGATGAAGAAGGATGATCA
GATTGCTGCT
GCTATTGCTTTGAGGGGGATGGCTAAGGATGGAAAGTTTGCTGTGAAGGATGGTGAGAAAGAGAAGGCTG
AGTATACTAT
TAAGGGAGCTGCTGAGTCTGCAGTTCGCAAAGTTTTAGG

>6
GCGATATAAGTAGTACGACGGGGAAACCAGATAGTACAGGTTCTGTTGGAACTGCCGTTGAGTATACTAT
TAAGGAAGTT
AGCGAGTTGTTGGATAAGCTGGTAAAAGCTGTAAAGACAGCTGAGGGGGCTTCAAGTGGTACTGCTGCAA
TTGGAGAAGT
TGTGGCTGATGCTGATGCTGCAAAGGTTGCTGATAAGGCGAGTGTGAAGGGGATTGCTAAGGGGATAAAG
GAGATTGTTG
AAGCTGCTGGGGGGAGTGAAAAGCTGAAAGCTGTTGCTGCTGCTAAAGGGGAGAATAATAAAGGGGCAGG
GAAGTTGTTT
GGGAAGGCTGGTGCTGCTGCTCATGGGGACAGTGAGGCTGCTAGCAAGGCGGCTGGTGCTGTTAGTGCTG
TTAGTGGGGA
GCAGATATTAAGTGCGATTGTTACGGCTGCTGATGCGGCTGAGCAGGATGGAAAGAAGCCTGAGGAGGCT
AAAAATCCGA
TTGCTGCTGCTATTGGGGATAAAGATGGGGGTGCGGAGTTTGGTCAGGATGAGATGAAGAAGGATGATCA
GATTGCTGCT
GCTATTGCTTTGAGGGGGATGGCTAAGGATGGAAAGTTTGCTGTGAAGGATGGTGAGAAAGAGAAGGCTG
AGTATACTAT
TAAGGGAGCTGCTGAGTCTGCAGTTCGCAAAGTTTTAGG

>7
GCGATATAAGTAGTACGACGGGGAAACCAGATAGTACAGGTTCTGTTGGAACTGCCGTTGAGTATACTAT
TAAGGAAGTT
AGCGAGTTGTTGGATAAGCTGGTAAAAGCTGTAAAGACAGCTGAGGGGGCTTCAAGTGGTACTGCTGCAA
TTGGAGAAGT
TGTGGCTGATGCTGATGCTGCAAAGGTTGCTGATAAGGCGAGTGTGAAGGGGATTGCTAAGGGGATAAAG
GAGATTGTTG
AAGCTGCTGGGGGGAGTGAAAAGCTGAAAGCTGTTGCTGCTGCTAAAGGGGAGAATAATAAAGGGGCAGG
GAAGTTGTTT
GGGAAGGCTGGTGCTGCTGCTCATGGGGACAGTGAGGCTGCTAGCAAGGCGGCTGGTGCTGTTAGTGCTG
TTAGTGGGGA
GCAGATATTAAGTGCGATTGTTACGGCTGCTGATGCGGCTGAGCAGGATGGAAAGAAGCCTGAGGAGGCT
AAAAATCCGA
TTGCTGCTGCTATTGGGGATAAAGATGGGGGTGCGGAGTTTGGTCAGGATGAGATGAAGAAGGATGATCA
GATTGCTGCT
GCTATTGCTTTGAGGGGGATGGCTAAGGATGGAAAGTTTGCTGTGAAGGATGGTGAGAAAGAGAAGGCTG
AGTATACTAT

TAAGGGAGCTGCTGAGTCTGCAGTTCGCAAAGTTTTAGG

>8
GCGATATAAGTAGTACGACGGGGAAACCAGATAGTACAGGTTCTGTTGGAACTGCCGTTGAGTATACTAT
TAAGGAAGTT
AGCGAGTTGTTGGATAAGCTGGTAAAAGCTGTAAAGACAGCTGAGGGGGCTTCAAGTGGTACTGCTGCAA
TTGGAGAAGT
TGTGGCTGATGCTGATGCTGCAAAGGTTGCTGATAAGGCGAGTGTGAAGGGGATTGCTAAGGGGATAAAG
GAGATTGTTG
AAGCTGCTGGGGGGAGTGAAAAGCTGAAAGCTGTTGCTGCTGCTAAAGGGGAGAATAATAAAGGGGCAGG
GAAGTTGTTT
GGGAAGGCTGGTGCTGCTGCTCATGGGGACAGTGAGGCTGCTAGCAAGGCGGCTGGTGCTGTTAGTGCTG
TTAGTGGGGA
GCAGATATTAAGTGCGATTGTTACGGCTGCTGATGCGGCTGAGCAGGATGGAAAGAAGCCTGAGGAGGCT
AAAAATCCGA
TTGCTGCTGCTATTGGGGATAAAGATGGGGGTGCGGAGTTTGGTCAGGATGAGATGAAGAAGGATGATCA
GATTGCTGCT
GCTATTGCTTTGAGGGGGATGGCTAAGGATGGAAAGTTTGCTGTGAAGGATGGTGAGAAAGAGAAGGCTG
AGTATACTAT
TAAGGGAGCTGCTGAGTCTGCAGTTCGCAAAGTTTTAGG

>9
GCGATATAAGTAGTACGACGGGGAAACCAGATAGTACAGGTTCTGTTGGAACTGCCGTTGAGTATACTAT
TAAGGAAGTT
AGCGAGTTGTTGGATAAGCTGGTAAAAGCTGTAAAGACAGCTGAGGGGGCTTCAAGTGGTACTGCTGCAA
TTGGAGAAGT
TGTGGCTGATGCTGATGCTGCAAAGGTTGCTGATAAGGCGAGTGTGAAGGGGATTGCTAAGGGGATAAAG
GAGATTGTTG
AAGCTGCTGGGGGGAGTGAAAAGCTGAAAGCTGTTGCTGCTGCTAAAGGGGAGAATAATAAAGGGGCAGG
GAAGTTGTTT
GGGAAGGCTGGTGCTGCTGCTCATGGGGACAGTGAGGCTGCTAGCAAGGCGGCTGGTGCTGTTAGTGCTG
TTAGTGGGGA
GCAGATATTAAGTGCGATTGTTACGGCTGCTGATGCGGCTGAGCAGGATGGAAAGAAGCCTGAGGAGGCT
AAAAATCCGA
TTGCTGCTGCTATTGGGGATAAAGATGGGGGTGCGGAGTTTGGTCAGGATGAGATGAAGAAGGATGATCA
GATTGCTGCT
GCTATTGCTTTGAGGGGGATGGCTAAGGATGGAAAGTTTGCTGTGAAGGATGGTGAGAAAGAGAAGGCTG
AGTATACTAT
TAAGGGAGCTGCTGAGTCTGCAGTTCGCAAAGTTTTAGG

>10
GCGATATAAGTAGTACGACGGGGAAACCAGATAGTACAGGTTCTGTTGGAACTGCCGTTGAGTATACTAT
TAAGGAAGTT
AGCGAGTTGTTGGATAAGCTGGTAAAAGCTGTAAAGACAGCTGAGGGGGCTTCAAGTGGTACTGCTGCAA
TTGGAGAAGT
TGTGGCTGATGCTGATGCTGCAAAGGTTGCTGATAAGGCGAGTGTGAAGGGGATTGCTAAGGGGATAAAG
GAGATTGTTG
AAGCTGCTGGGGGGAGTGAAAAGCTGAAAGCTGTTGCTGCTGCTAAAGGGGAGAATAATAAAGGGGCAGG
GAAGTTGTTT
GGGAAGGCTGGTGCTGCTGCTCATGGGGACAGTGAGGCTGCTAGCAAGGCGGCTGGTGCTGTTAGTGCTG

TTAGTGGGGA
GCAGATATTAAGTGCGATTGTTACGGCTGCTGATGCGGCTGAGCAGGATGGAAAGAAGCCTGAGGAGGCT
AAAAATCCGA
TTGCTGCTGCTATTGGGGATAAAGATGGGGGTGCGGAGTTTGGTCAGGATGAGATGAAGAAGGATGATCA
GATTGCTGCT
GCTATTGCTTTGAGGGGGATGGCTAAGGATGGAAAGTTTGCTGTGAAGGATGGTGAGAAAGAGAAGGCTG
AGTATACTAT
TAAGGGAGCTGCTGAGTCTGCAGTTCGCAAAGTTTTAGG

>11
GCGATATAAGTAGTACGACGGGGAAACCAGATAGTACAGGTTCTGTTGGAACTGCCGTTGAGTATACTAT
TAAGGAAGTT
AGCGAGTTGTTGGATAAGCTGGTAAAAGCTGTAAAGACAGCTGAGGGGGCTTCAAGTGGTACTGCTGCAA
TTGGAGAAGT
TGTGGCTGATGCTGATGCTGCAAAGGTTGCTGATAAGGCGAGTGTGAAGGGGATTGCTAAGGGGATAAAG
GAGATTGTTG
AAGCTGCTGGGGGGAGTGAAAAGCTGAAAGCTGTTGCTGCTGCTAAAGGGGAGAATAATAAAGGGGCAGG
GAAGTTGTTT
GGGAAGGCTGGTGCTGCTGCTCATGGGGACAGTGAGGCTGCTAGCAAGGCGGCTGGTGCTGTTAGTGCTG
TTAGTGGGGA
GCAGATATTAAGTGCGATTGTTACGGCTGCTGATGCGGCTGAGCAGGATGGAAAGAAGCCTGAGGAGGCT
AAAAATCCGA
TTGCTGCTGCTATTGGGGATAAAGATGGGGGTGCGGAGTTTGGTCAGGATGAGATGAAGAAGGATGATCA
GATTGCTGCT
GCTATTGCTTTGAGGGGGATGGCTAAGGATGGAAAGTTTGCTGTGAAGGATGGTGAGAAAGAGAAGGCTG
AGTATACTAT
TAAGGGAGCTGCTGAGTCTGCAGTTCGCAAAGTTTTAGG

>12
GCGATATAAGTAGTACGACGGGGAAACCAGATAGTACAGGTTCTGTTGGAACTGCCGTTGAGTATACTAT
TAAGGAAGTT
AGCGAGTTGTTGGATAAGCTGGTAAAAGCTGTAAAGACAGCTGAGGGGGCTTCAAGTGGTACTGCTGCAA
TTGGAGAAGT
TGTGGCTGATGCTGATGCTGCAAAGGTTGCTGATAAGGCGAGTGTGAAGGGGATTGCTAAGGGGATAAAG
GAGATTGTTG
AAGCTGCTGGGGGGAGTGAAAAGCTGAAAGCTGTTGCTGCTGCTAAAGGGGAGAATAATAAAGGGGCAGG
GAAGTTGTTT
GGGAAGGCTGGTGCTGCTGCTCATGGGGACAGTGAGGCTGCTAGCAAGGCGGCTGGTGCTGTTAGTGCTG
TTAGTGGGGA
GCAGATATTAAGTGCGATTGTTACGGCTGCTGATGCGGCTGAGCAGGATGGAAAGAAGCCTGAGGAGGCT
AAAAATCCGA
TTGCTGCTGCTATTGGGGATAAAGATGGGGGTGCGGAGTTTGGTCAGGATGAGATGAAGAAGGATGATCA
GATTGCTGCT
GCTATTGCTTTGAGGGGGATGGCTAAGGATGGAAAGTTTGCTGTGAAGGATGGTGAGAAAGAGAAGGCTG
AGTATACTAT
TAAGGGAGCTGCTGAGTCTGCAGTTCGCAAAGTTTTAGG

>13
GCGATATAAGTAGTACGACGGGGAAACCAGATAGTACAGGTTCTGTTGGAACTGCCGTTGAGTATACTAT
TAAGGAAGTT

AGCGAGTTGTTGGATAAGCTGGTAAAAGCTGTAAAGACAGCTGAGGGGGCTTCAAGTGGTACTGCTGCAA
TTGGAGAAGT
TGTGGCTGATGCTGATGCTGCAAAGGTTGCTGATAAGGCGAGTGTGAAGGGGATTGCTAAGGGGATAAAG
GAGATTGTTG
AAGCTGCTGGGGGGAGTGAAAAGCTGAAAGCTGTTGCTGCTGCTAAAGGGGAGAATAATAAAGGGGCAGG
GAAGTTGTTT
GGGAAGGCTGGTGCTGCTGCTCATGGGGACAGTGAGGCTGCTAGCAAGGCGGCTGGTGCTGTTAGTGCTG
TTAGTGGGGA
GCAGATATTAAGTGCGATTGTTACGGCTGCTGATGCGGCTGAGCAGGATGGAAAGAAGCCTGAGGAGGCT
AAAAATCCGA
TTGCTGCTGCTATTGGGGATAAAGATGGGGGTGCGGAGTTTGGTCAGGATGAGATGAAGAAGGATGATCA
GATTGCTGCT
GCTATTGCTTTGAGGGGGATGGCTAAGGATGGAAAGTTTGCTGTGAAGGATGGTGAGAAAGAGAAGGCTG
AGTATACTAT
TAAGGGAGCTGCTGAGTCTGCAGTTCGCAAAGTTTTAGG

>14
GCGATATAAGTAGTACGACGGGGAAACCAGATAGTACAGGTTCTGTTGGAACTGCCGTTGAGTATACTAT
TAAGGAAGTT
AGCGAGTTGTTGGATAAGCTGGTAAAAGCTGTAAAGACAGCTGAGGGGGCTTCAAGTGGTACTGCTGCAA
TTGGAGAAGT
TGTGGCTGATGCTGATGCTGCAAAGGTTGCTGATAAGGCGAGTGTGAAGGGGATTGCTAAGGGGATAAAG
GAGATTGTTG
AAGCTGCTGGGGGGAGTGAAAAGCTGAAAGCTGTTGCTGCTGCTAAAGGGGAGAATAATAAAGGGGCAGG
GAAGTTGTTT
GGGAAGGCTGGTGCTGCTGCTCATGGGGACAGTGAGGCTGCTAGCAAGGCGGCTGGTGCTGTTAGTGCTG
TTAGTGGGGA
GCAGATATTAAGTGCGATTGTTACGGCTGCTGATGCGGCTGAGCAGGATGGAAAGAAGCCTGAGGAGGCT
AAAAATCCGA
TTGCTGCTGCTATTGGGGATAAAGATGGGGGTGCGGAGTTTGGTCAGGATGAGATGAAGAAGGATGATCA
GATTGCTGCT
GCTATTGCTTTGAGGGGGATGGCTAAGGATGGAAAGTTTGCTGTGAAGGATGGTGAGAAAGAGAAGGCTG
AGTATACTAT
TAAGGGAGCTGCTGAGTCTGCAGTTCGCAAAGTTTTAGG

>15
GCGATATAAGTAGTACGACGGGGAAACCAGATAGTACAGGTTCTGTTGGAACTGCCGTTGAGTATACTAT
TAAGGAAGTT
AGCGAGTTGTTGGATAAGCTGGTAAAAGCTGTAAAGACAGCTGAGGGGGCTTCAAGTGGTACTGCTGCAA
TTGGAGAAGT
TGTGGCTGATGCTGATGCTGCAAAGGTTGCTGATAAGGCGAGTGTGAAGGGGATTGCTAAGGGGATAAAG
GAGATTGTTG
AAGCTGCTGGGGGGAGTGAAAAGCTGAAAGCTGTTGCTGCTGCTAAAGGGGAGAATAATAAAGGGGCAGG
GAAGTTGTTT
GGGAAGGCTGGTGCTGCTGCTCATGGGGACAGTGAGGCTGCTAGCAAGGCGGCTGGTGCTGTTAGTGCTG
TTAGTGGGGA
GCAGATATTAAGTGCGATTGTTACGGCTGCTGATGCGGCTGAGCAGGATGGAAAGAAGCCTGAGGAGGCT
AAAAATCCGA
TTGCTGCTGCTATTGGGGATAAAGATGGGGGTGCGGAGTTTGGTCAGGATGAGATGAAGAAGGATGATCA
GATTGCTGCT

GCTATTGCTTTGAGGGGGATGGCTAAGGATGGAAAGTTTGCTGTGAAGGATGGTGAGAAAGAGAAGGCTG
AGTATACTAT
TAAGGGAGCTGCTGAGTCTGCAGTTCGCAAAGTTTTAGG
