# Supplementary material for: Breaking a barrier: In trans vlsE recombination and genetic manipulation of the native vlsE gene of the Lyme disease pathogen
Source: PLoS Pathog. 2025 Jan 10;21(1):e1012871. doi: 10.1371/journal.ppat.1012871 (PMC11756760; doi:10.1371/journal.ppat.1012871)
Supplement: S6 Fig — Complete DNA sequences of native vlsE on lp28-1 used in multiple sequence alignment in Fig 6B in DR mutant group. The alignment spans the positions 1–660, starting at the vlsE sequence where primer P243 binds the N-terminal constant region and ending at 16 bp of the C-terminal constant region. (PDF) [file ppat.1012871.s006.pdf]

>vlsE
GCGATATAAGTAGTACGACGGGGAAACCAGATAGTACAGGTTCTGTTGGAACTGCCGTTGAGGGGGCTAT
TAAGGAAGTT
AGCGAGTTGTTGGATAAGCTGGTAAAAGCTGTAAAGACAGCTGAGGGGGCTTCAAGTGGTACTGCTGCAA
TTGGAGAAGT
TGTGGCTGATGCTGATGCTGCAAAGGTTGCTGATAAGGCGAGTGTGAAGGGGATTGCTAAGGGGATAAAG
GAGATTGTTG
AAGCTGCTGGGGGGAGTGAAAAGCTGAAAGCTGTTGCTGCTGCTAAAGGGGAGAATAATAAAGGGGCAGG
GAAGTTGTTT
GGGAAGGCTGGTGCTGCTGCTCATGGGGACAGTGAGGCTGCTAGCAAGGCGGCTGGTGCTGTTAGTGCTG
TTAGTGGGGA
GCAGATATTAAGTGCGATTGTTACGGCTGCTGATGCGGCT------
GAGCAGGATGGAAAGAAGCCTGAGGAGGCTAAAA
ATCCGATTGCTGCTGCTATTGGGGATAAAGATGGGGGTGCG------
GAGTTTGGTCAGGATGAGATGAAGAAGGATGAT
CAGATTGCTGCTGCTATTGCTTTGAGGGGGATGGCTAAGGATGGAAAGTTTGCTGTGAAGGATGGTGAGA
AA-GAGAAGG
CTGAGGGGGCTATTAAGGGAGCTGCTGAGTCTGCAGTTCGCAAAGTTTTAGGG

>1
GCGATATAAGTAGTACGACGGGGAAACCAGATAGTACAGGTTCTGTTGGAACTGCCGTTGAGGGGGCTAT
TAAGGAAGTT
AGCGAGTTGTTGGATAAGCTGGTAAAAGCTGTAAAGACAGCTGAGGGGGCTTCAAGTGGTACTGCTGCAA
TTGGAGAAGT
TGTGGCTGATGCTGATGCTGCAAAGGTTGCTGATAAGGCGAGTGTGAAGGGGATTGCTAAGGGGATAAAG
GAGATTGTTG
AAGCTGCTGGGGGGAGTGAAAAGCTGAAAGCTGTTGCTGCTGCTAAAGGGGAGAATAATAAAGGGGCAGG
GAAGTTGTTT
GGGAAGGCTGGTGCTGCTGCTCATGGGGACAGTGAGGCTGCTAGCAAGGCGGCTGGTGCTGTTAGTGCTG
TTAGTGGGGA
GCAGATATTAAGTGCGATTGTTACGGCTGCTGATGCGGCT------
GAGCAGGATGGAAAGAAGCCTGAGGAGGCTAAAA
ATCCGATTGCTGCTGCTATTGGGGATAAAGATGGGGGTGCG------
GAGTTTGGTCAGGATGAGATGAAGAAGGATGAT
CAGATTGCTGCTGCTATTGCTTTGAGGGGGATGGCTAAGGATGGAAAGTTTGCTGTGAAGGATGGTGAGA
AAAGAGAAGG
CTGAGTATACTATTAAGGGAGCTGCTGAGTCTGCAGTTCGCAAAGTTTTAGGG

>2
GCGATATAAGTAGTACGACGGGGAAACCAGATAGTACAGGTTCTGTTGGAACTGCCGTTGAGGGGGCTAT
TAAGGAAGTT
AGCGAGTTGTTGGATAAGCTGGTAAAAGCTGTAAAGACAGCTGAGGGGGCTTCAAGTGGTACTGCTGCAA
TTGGAGAAGT
TGTGGCTGATGCTGATGCTGCAAAGGTTGCTGATAAGGCGAGTGTGAAGGGGATTGCTAAGGGGATAAAG
GAGATTGTTG
AAGCTGCTGGGGGGAGTGAAAAGCTGAAAGCTGTTGCTGCTGCTAAAGGGGAGAATAATAAAGGGGCAGG
GAAGTTGTTT
GGGAAGGCTGGTGCTGCTGCTCATGGGGACAGTGAGGCTGCTAGCAAGGCGGCTGGTGCTGTTAGTGCTG
TTAGTGGGGA
GCAGATATTAAGTGCGATTGTTACGGCTGCTGATGCGGCT------

GAGCAGGATGGAAAGAAGCCTGAGGAGGCTAAAA
ATCCGATTGCTGCTGCTATTGGGGATAAAGATGGGGGTGCG------
GAGTTTGGTCAGGATGAGATGAAGAAGGATGAT
CAGATTGCTGCTGCTATTGCTTTGAGGGGGATGGCTAAGGATGGAAAGTTTGCTGTGAAGGATGGTGAGA
AAGAAGAAGG
CTGAGTATACTATTAAGGGAGCTGCTGAGTCTGCAGTTCGCAAAGTTTTAGGG

>3
GCGATATAAGTAGTACGACGGGGAAACCAGATAGTACAGGTTCTGTTGGAACTGCCGTTGAGGGGGCTAT
TAAGGAAGTT
AGCGAGTTGTTGGATAAGCTGGTAAAAGCTGTAAAGACAGCTGAGGGGGCTTCAAGTGGTACTGCTGCAA
TTGGAGAAGT
TGTGGCTGATGCTGATGCTGCAAAGGTTGCTGATAAGGCGAGTGTGACGGGGATTGCTAAGGGGATAAAG
GAGATTGTTG
AAGCTGCTGGGGGGAGTGAAAAGCTGAAAGCTGTTGCTGCTGCTACAGGGGAGAATAATAAAGGGGCAGG
GAAGTTGTTT
GGGAAGGCTGGTGCTAATGCTCATGGGGACAGTGAGGCTGCTAGCAAGGCGGCTGGTGCTGTTAGTGCTG
TTAGTGGGGA
GCAGATATTAAGTGCGATTGTTAAGGCTGCTGATGCGGCTGCTGGTGATCAGGAGGGAAAGAAGCCTGGG
GATGCTAAAA
ATCCGATTGCTGCTGCTATTGGGAAGGGTAATGAGGAGAATGGTGCGGAGTTTGGTCAGGATGAGATGAA
GAAGGATGAT
CAGATTGCTGCTGCTATTGCTTTGAGGGGGATGGCTAAGGATGGAAAGTTTGCTGTGAAGAGTGGNGAGA
AAGAAGAAGG
CTGAGGGGGCTATTAAGGGAGCTGCTGAGTCTGCAGTTCGCAAAGTTTTAGGG

>4
GCGATATAAGTAGTACGACGGGGAAACCAGATAGTACAGGTTCTGTTGGAACTGCCGTTGAGGGGGCTAT
TAAGGAAGTT
AGCGAGTTGTTGGATAAGCTGGTAAAAGCTGTAAAGACAGCTGAGGGGGCTTCAAGTGGTACTGCTGCAA
TTGGAGAAGT
TGTGGCTGATGATGCTGCTGCGAAGGCTGCTGATAAGGCGAGTGTGACGGGGATTGCTAAGGGGATAAAG
GAGATTGTTG
AAGCTGCTGGGGGGAGTGAAAAGCTGAAAGCTGTTGCTGCTGCTAAAGGGGAGAATAATAAAGGGGCAGG
GAAGTTGTTT
GGGAAGGCTGGTGCTGCTGCTCATGGGGACAGTGAGGCTGCTAGCAAGGCGGCTGGTGCTGTTAGTGCTG
TTAGTGGGGA
GCAGATATTAAGTGCGATTGTTACGGCTGCTGATGCGGCT------
GAGCAGGATGGAAAGAAGCCTGAGGAGGCTAAAA
ATCCGATTGCTGCTGCTATTGGGGATAAAGATGGGGGTGCG------
GAGTTTGGTCAGGATGAGATGAAGAAGGATGAT
CAGATTGCTGCTGCTATTGCTTTGAGGGGGATGGCTAAGGATGGAAAGTTTGCTGTGAAGGATGGTGAGA
A-AGAGAAGG
CTGAGTATACTATTAAGGGAGCTGCTGAGTCTGCAGTTCGCAAAGTTTTAGGG

>5
GCGATATAAGTAGTACGACGGGGAAACCAGATAGTACAGGTTCTGTTGGAACTGCCGTTGAGGGGGCTAT
TAAGGAAGTT
AGCGAGTTGTTGGATAAGCTGGTAAAAGCTGTAAAGACAGCTGAGGGGGCTTCAAGTGGTACTGCTGCAA
TTGGAGAAGT

TGTGGCTGATGCTGATGCTGCAAAGGTTGCTGATAAGGCGAGTGTGAAGGGGATTGCTAAGGGGATAAAG
GAGATTGTTG
AAGCTGCTGGGGGGAGTGAAAAGCTGAAAGCTGTTGCTGCTGCTAAAGGGGAGAATAATAAAGGGGCAGG
GAAGTTGTTT
GGGAAGGCTGGTGCTGCTGCTCATGGGGACAGTGAGGCTGCTAGCAAGGCGGCTGGTGCTGTTAGTGCTG
TTAGTGGGGA
GCAGATATTAAGTGCGATTGTTACGGCTGCTGATGCGGCT------
GAGCAGGATGGAAAGAAGCCTGAGGAGGCTAAAA
ATCCGATTGCTGCTGCTATTGGGGATAAAGATGGGGGTGCG------
GAGTTTGGTCAGGATGAGATGAAGAAGGATGAT
CAGATTGCTGCTGCTATTGCTTTGAGGGGGATGGCTAAGGATGGAAAGTTTGCTGTGAAGGATGGTGAGA
AAAGAGAAGG
CTGAGTATACTATTAAGGGAGCTGCTGAGTCTGCAGTTCGCAAAGTTTTAGGG

>6
GCGATATAAGTAGTACGACGGGGAAACCAGATAGTACAGGTTCTGTTGGAACTGCCGTTGAGTATACTAT
TAAGGAAGTT
AGCGAGTTGTTGGATAAGCTGGTAAAAGCTGTAAAGACAGCTGAGGGGGCTTCAAGTGGTACTGCTGCAA
TTGGAGAAGT
TGTGGATAA------
TGCTGCGAAGGCTGCTGATAAGGCGAGTGTGACGGGGATTGCTAAGGGGATAAAGGAGATTGTTG
AAGCTGCTGGGGGGAGTGAAAAGCTGAAAGTTGCTG---
CTGCTACAGGGGAGAGTAATAAAGGGGCAGGGAAGTTGTTT
GGGAAGGCTGGTGCTGATGCTCATGGGGACAGTGAGGCTGCTAGCAAGGCGGCTGGTGCTGTTAGTGCTG
TTAGTGGGGA
GCAGATATTAAGTGCGATTGTTACGGCTGCTGATGCGGCT------
GAGCAGGATGGAAAGAAGCCTGAGGAGGCTAAAA
ATCCGATTGCTGCTGCTATTGGGGATAAAGATGGGGGTGCG------
GAGTTTGGTCAGGATGAGATGAAGAAGGATGAT
CAGATTGCTGCTGCTATTGCTTTGAGGGGGATGGCTAAGGATGGAAAGTTTGCTGTGAAGGATGGTGAGA
A-AGAGAAGG
CTGAGTATACTATTAAGGGAGCTGCTGAGTCTGCAGTTCGCAAAGTTTTAGGG

>7
GCGATATAAGTAGTACGACGGGGAAACCAGATAGTACAGGTTCTGTTGGAACTGCCGTTGAGGGGGCTAT
TAAGGAAGTT
AGCGAGTTGTTGGATAAGCTGGTAAAAGCTGTAAAGACAGCTGAGGGGGCTTCAAGTGGTACTGCTGCAA
TTGGAGAAGT
TGTGGCTGATGATGCTGCTGCGAAGGCTGCTGATAAGGCGAGTGTGACGGGGATTGCTAAGGGGATAAAG
GAGATTGTTG
AAGCTGCTGGGGGGAGTGAAAAGCTGAAAGCTGTTGCTGCTGCTAAAGGGGAGAATAATAAAGGGGCAGG
GAAGTTGTTT
GGGAAGGCTGGTGCTGCTGCTCATGGGGACAGTGAGGCTGCTAGCAAGGCGGCTGGTGCTGTTAGTGCTG
TTAGTGGGGA
GCAGATATTAAGTGCGATTGTTACGGCTGCTGATGCGGCT------
GAGCAGGATGGAAAGAAGCCTGAGGAGGCTAAAA
ATCCGATTGCTGCTGCTATTGGGGATAAAGATGGGGGTGCG------
GAGTTTGGTCAGGATGAGATGAAGAAGGATGAT
CAGATTGCTGCTGCTATTGCTTTGAGGGGGATGGCTAAGGATGGAAAGTTTGCTGTGAAGGATGGTGAGA
A-AGAGAAGG

CTGAGTATACTATTAAGGGAGCTGCTGAGTCTGCAGTTCGCAAAGTTTTAGGG

>8
GCGATATAAGTAGTACGACGGGGAAACCAGATAGTACAGGTTCTGTTGGAACTGCCGTTGAGGGGGCTAT
TAAGGAAGTT
AGCGAGTTGTTGGATAAGCTGGTAAAAGCTGTAAAGACAGCTGAGGGGGCTTCAAGTGGTACTGCTGCAA
TTGGAGAAGT
TGTGGCTGATGCTGATGCTGCAAAGGTTGCTGATAAGGCGAGTGTGACGGGGATTGCTAAGGGGATAAAG
GAGATTGTTG
AAGCTGCTGGGGGGAGTGAAAAGCTGAAAGCTGTTGCTGCTGCTACAGGGGAGAATAATAAAGGGGCAGG
GAAGTTGTTT
GGGAAGGCTGGTGCTAATGCTCATGGGGACAGTGAGGCTGCTAGCAAGGCGGCTGGTGCTGTTAGTGCTG
TTAGTGGGGA
GCAGATATTAAGTGCGATTGTTAAGGCTGCTGATGCGGCTG------
AGCAGGATGGAAAGAAGCCTGCAGATGCTACAA
ATCCGATTGCTGCTGCTATTGGGAAGGGTGATGCGGAGAATGGTGCGGAGTTTGGTAAGGATGAGATGAA
GAAGGATGAT
CAGATTGCTGCTGCTATTGCTTTGAGGGGGATGGCTAAGGATGGAAAGTTTGCTGTGAAGAGTGGTGAGA
AAGA-GAAGG
CTGAGGGGGCTATTAAGGGAGCTGCTGAGTCTGCAGTTCGCAAAGTTTTAGGG

>9
GCGATATAAGTAGTACGACGGGGAAACCAGATAGTACAGGTTCTGTTGGAACTGCCGTTGAGGGGGCTAT
TAAGGAAGTT
AGCGAGTTGTTGGATAAGCTGGTAAAAGCTGTAAAGACAGCTGAGGGGGCTTCAAGTGGTACTGCTGCAA
TTGGAGAAGT
TGTGGATAAT------
GCTGCGAAGGCTGCTGATAAGGATAGTGTGAAGGGGATTGCTAAGGGGATAAAGGAGATTGTTG
AAGCTGCTGGGGGGAGTGAAAAGCTGAAAGCTGTTGCTGCTGCTACAGGGGAGAATAATAAAGGGGCAGG
GAAGTTGTTT
GGGAAGGCTGGTGCTGATGCTAATGGGGACAGTGAGGCTGCTAGCAAGGCGGCTGGTGCTGTTAGTGCTG
TTAGTGGGGA
GCAGATATTAAGTGCGATTGTTAAGGCTGCTGGTGAGGCT------
GATCAGGAGGGAAAGAAGCCTGAGGAGGCTAAAA
ATCCGATTGCTGCTGCTATTGGGAAGGGTGATGCGGATGATG---
GTGCGGAGTTTGATCATGAGATGAAGAAGGATGAT
CAGATTGCTGCTGCTATTGCTTTGAGGGGGATGGCTAAGGATGGAAAGTTTGCTGTGAAGGATGGTGAGA
A-NGNCAAGG
CTGAGTATACTATTAAGGGAGCTGCTGAGTCTGCAGTTCGCAAAGTTTTAGGG

>10
GCGATATAAGTAGTACGACGGGGAAACCAGATAGTACAGGTTCTGTTGGAACTGCCGTTGAGGGGGCTAT
TAAGGAAGTT
AGCGAGTTGTTGGATAAGCTGGTAAAAGCTGTAAAGACAGCTGAGGGGGCTTCAAGTGGTACTGCTGCAA
TTGGAGAAGT
TGTGGATAA------
TGATGCTAAGGTTGCTGATAAGGATAGTGTGACGGGGATTGCTAAGGGGATAAAGGAGATTGTTG
AAGCTGCTGGGGGGAGTGAAAAGCTGAAAGTTGCTG---
CTGCTACAGGGGAGAATAATAAAGGGGCAGGGAAGTTGTTT
GGGAAGGCTGGTGCTGATGCTAATGGGGACAGTGAGGCTGCTAGCAAGGCGGCTGGTGCTGTTAGTGCTG

TTAGTGGGGA
GCAGATATTAAGTGCGATTGTTACGGCTGCTGATGCGGCT------
GATCAGGATGGAAAGAAGCCTGAGGAGGCTAAAA
ATCCGATTGCTGCTGCTATTGGGGATAAAGATGGGGGTGCG------
GAGTTTGGTCAGGATGAGATGAAGAAGGATGAT
CAGATTGCTGCTGCTATTGCTTTGAGGGGGATGGCTAAGGATGGAAAGTTTGCTGTGAAGGATGGTGAGA
A-AGAGAAGG
CTGAGTATACTATTAAGGGAGCTGCTGAGTCTGCAGTTCGCAAAGTTTTAGGG

>11
GCGATATAAGTAGTACGACGGGGAAACCAGATAGTACAGGTTCTGTTGGAACTGCCGTTGAGGGGGCTAT
TAAGGAAGTT
AGCGAGTTGTTGGATAAGCTGGTAAAAGCTGTAAAGACAGCTGAGGGGGCTTCAAGTGGTACTGCTGCAA
TTGGAGAAGT
TGTGGATAA------
TGCTGCGAAGGCTGCTGATAAGGATAGTGTGACGGGGATTGCTAAGGGGATAAAGGAGATTGTTG
AAGCTGCTGGGGGGAGTGAAAAGCTGAAAGTTGCTG---CTGCTAAAGAGGG---
CAATGAAAAGGCAGGGAAGTTGTTT
GGGAAGGCTGGTGCTAATGCTCATGGGGACAGTGAGGCTGCTAGCAAGGCGGCTGGTGCTGTTAGTGCTG
TTAGTGGGGA
GCAGATATTAAGTGCGATTGTTACGGCTGCTGATGCGGCT------
GAGCAGGATGGAAAGAAGCCTGAGGAGGCTAAAA
ATCCGATTGCTGCTGCTATTGGGGATAAAGATGGGGGTGCG------
GAGTTTGGTCAGGATGAGATGAAGAAGGATGAT
CAGATTGCTGCTGCTATTGCTTTGAGGGGGATGGCTAAGGATGGAAAGTTTGCTGTGAAGGATGGTGAGA
A-AGAGAAGG
CTGAGTATACTATTAAGGGAGCTGCTGAGTCTGCAGTTCGCAAAGTTTTAGGG

>12
GCGATATAAGTAGTACGACGGGGAAACCAGATAGTACAGGTTCTGTTGGAACTGCCGTTGAGTATACTAT
TAAGGAAGTT
AGCGAGTTGTTGGATAAGCTGGTAAAAGCTGTAAAGACAGCTGAGGGGGCTTCAAGTGGTACTGCTGCAA
TTGGAGAAGT
TGTGGCTGATGCTGATGCTGCAAAGGTTGCTGATAAGGCGAGTGTGACGGGGATTGCTAAGGGGATAAAG
GAGATTGTTG
AAGCTGCTGGGGGGAGTGAAAAGCTGAAAGCTGTTGCTGCTGCTACAGGGGAGAATAATAAAGGGGCAGG
GAAGTTGTTT
GGGAAGGCTGGTGCTAATGCTCATGGGGACAGTGAGGCTGCTAGCAAGGCGGCTGGTGCTGTTAGTGCTG
TTAGTGGGGA
GCAGATATTAAGTGCGATTGTTAAGGCTGCTGGTGCGGCTG------
AGCAGGAGGGAAAGAAGCCTGAGGAGGCTAAAA
ATCCGATTGCTGCTGCTATTGGGGAGGGTAATGAGGAGAATGGTGCGGATTTTGGTAAGGATGAGATGAA
GAAGGATGAT
CAGATTGCTGCTGCTATTGCTTTGAGGGGGATGGCTAAGGATGGAAAGTTTGCTGTGAAGAGTGGTGAGA
AAGG-GAAGG
CTGAGGGGGCTATTAAGGGAGCTGCTGAGTCTGCAGTTCGCAAAGTTTTAGGG

>13
GCGATATAAGTAGTACGACGGGGAAACCAGATAGTACAGGTTCTGTTGGAACTGCCGTTGAGGGGGCTAT
TAAGGAAGTT

AGCGAGTTGTTGGATAAGCTGGTAAAAGCTGTAAAGACAGCTGAGGGGGCTTCAAGTGGTACTGCTGCAA
TTGGAGAAGT
TGTGGCTGATGCTGATGCTGCAAAGGTTGCTGATAAGGCGAGTGTGAAGGGGATTGCTAAGGGGATAAAG
GAGATTGTTG
AAGCTGCTGGGGGGAGTGAAAAGCTGAAAGCTGTTGCTGCTGCTAAAGGGGAGAATAATAAAGGGGCAGG
GAAGTTGTTT
GGGAAGGCTGGTGCTGCTGCTCATGGGGACAGTGAGGCTGCTAGCAAGGCGGCTGGTGCTGTTAGTGCTG
TTAGTGGGGA
GCAGATATTAAGTGCGATTGTTACGGCTGCTGATGCGGCT------
GAGCAGGATGGAAAGAAGCCTGGGGATGCTAAAA
ATCCGATTGCTGCTGCTATTGGGAAGGGTAATGCGGATGATGGTGCGGAGTTT---
AAGGATGGGATGAAGAAGGATGAT
CAGATTGCTGCTGCTATTGCTTTGAGGGGGATGGCTAAGGATGGAAAGTTTGCTGTGAAGAAGGATGAGA
AA-GGGAAGG
CTGAGGGGGCTATTAAGGGAGCTGCTGAGTCTGCAGTTCGCAAAGTTTTAGGG

>14
GCGATATAAGTAGTACGACGGGGAAACCAGATAGTACAGGTTCTGTTGGAACTGCCGTTGAGGGGGCTAT
TAAGGAAGTT
AGCGAGTTGTTGGATAAGCTGGTAAAAGCTGTAAAGACAGCTGAGGGGGCTTCAAGTGGTACTGCTGCAA
TTGGAGAAGT
TGTGGCTGATGCTGATGCTGCAAAGGTTGCTGATAAGGCGAGTGTGACGGGGATTGCTAAGGGGATAAAG
GAGATTGTTG
AAGCTGCTGGGGGGAGTGAAAAGCTGAAAGCTGTTGCTGCTGCTACAGGGGAGAATAATAAAGGGGCAGG
GAAGTTGTTT
GGGAAGGCTGGTGCTAATGCTCATGGGGACAGTGAGGCTGCTAGCAAGGCGGCTGGTGCTGTTAGTGCTG
TTAGTGGGGA
GCAGATATTAAGTGCGATTGTTAAGGCTGCTGATGCGGCTGCTGGTGATCAGGAGGGAAAGAAGCCTGGG
GATGCTAAAA
ATCCGATTGCTGCTGCTATTGGGAAGGGTAATGAGGAGAATGGTGCGGAGTTTGGTCAGGATGAGATGAA
GAAGGATGAT
CAGATTGCTGCTGCTATTGCTTTGAGGGGGATGGCTAAGGATGGAAAGTTTGCTGTGAAGAGTGGNGAGA
AAGAAGAAGG
CTGAGGGGGCTATTAAGGGAGCTGCTGAGTCTGCAGTTCGCAAAGTTTTAGGG

>15
GCGATATAAGTAGTACGACGGGGAAACCAGATAGTACAGGTTCTGTTGGAACTGCCGTTGAGGGGGCTAT
TAAGGAAGTT
AGCGAGTTGTTGGATAAGCTGGTAAAAGCTGTAAAGACAGCTGAGGGGGCTTCAAGTGGTACTGCTGCAA
TTGGAGAAGT
TGTGGCTGATGCTGATGCTGCAAAGGTTGCTGATAAGGCGAGTGTGAAGGGGATTGCTAAGGGGATAAAG
GAGATTGTTG
AAGCTGCTGGGGGGAGTGAAAAGCTGAAAGCTGTTGCTGCTGCTAAAGGGGAGAATAATAAAGGGGCAGG
GAAGTTGTTT
GGGAAGGCTGGTGCTGCTGCTCATGGGGACAGTGAGGCTGCTAGCAAGGCGGCTGGTGCTGTTAGTGCTG
TTAGTGGGGA
GCAGATATTAAGTGCGATTGTTACGGCTGCTGATGCGGCT------
GAGCAGGATGGAAAGAAGCCTGGGGATGCTAAAA
ATCCGATTGCTGCTGCTATTGGGAAGGGTAATGCGGATGATGGTGCGGAGTTT---
AAGGATGGGATGAAGAAGGATGAT

CAGATTGCTGCTGCTATTGCTTTGAGGGGGATGGCTAAGGATGGAAAGTTTGCTGTGAAGAAGGATGAGA
AA–GGGAAGG
CTGAGGGGGCTATTAAGGGAGCTGCTGAGTCTGCAGTTCGCAAAGTTTTAGGG
