# Supplementary material for: Breaking a barrier: In trans vlsE recombination and genetic manipulation of the native vlsE gene of the Lyme disease pathogen
Source: PLoS Pathog. 2025 Jan 10;21(1):e1012871. doi: 10.1371/journal.ppat.1012871 (PMC11756760; doi:10.1371/journal.ppat.1012871)
Supplement: S1 Table — (DOCX) [file ppat.1012871.s008.docx]

**Table S1- Oligos used in the study**

| **Primer** | **Sequence (5’-3’)** | **Application** |
| --- | --- | --- |
| P243 | GCGATATAAGTAGTACGACGGGGAAACCAG | Amplification of *vlsE* central cassette region binding to N- and C-terminal constant regions |
| P244 | CAAGGCAGGAGGTGTTTCTTTACTAGCAGC |  |
| P338 | CCTCCCATGGGCCATTCTTTGGTGAATAAAC | Amplification of *pncA* along with 360 bp up- and 100 bp down-stream regions at NcoI and FseI restriction sites |
| P339 | CCTCGGCCGGCCCACAATATGGTAAACAATTTTCG |  |
| P257 | TACTATTAAGGAAGTTAGCGAGTTGTT | Forward and reverse primers for inverse PCR generating 17 bp DR mutation in DR1 region |
| P258 | TACTCAACGGCAGTTCCAACAGA |  |
| P280 | TACTATTAAGGGAGCTGCTGAGTCTGC | Forward and reverse primers for inverse PCR generating 17 bp DR mutation in DR2 region |
| P281 | TACTCAGCCTTCTCTTTCTCACCATC |  |
| P1350 | CTTATACTTTTCATTATAAGGAGACGATG | *vlsE* primers and probe used in ddPCR |
| P1351 | GCCTCTGCTACTAACCCAC |  |
| P1352 | HEX-CCAAGTTGCTGATAAGGACGACCCAAC-BHQ1 |  |
| P199 | TTG CTG ATC AAGCTC AAT ATA ACC A | *flaB* primers and probe used in ddPCR |
| P200 | TTG AGA CCC TGAAAG TGA TGC |  |
| P201 | 6FAM- AGCTGAAGAGCTTGGAATGCAGCCT- TAMRA |  |
| P1162 | AAATCTGATGTAAAAACCTATTTTACTACTGTAGCTGC | *vlsE* probe synthesis for Southern hybridization |
| P1163 | TTCTTTACTAGCAGCCTTCACTGAATCACC |  |
| P54 | CATATGAGCCATATTCAACGGGAAACG | kanamycin probe synthesis for Southern hybridization |
| P55 | AAAGCCGTTTCTGTAATGAAGGAG |  |
| P91 | CGCAGCAGCAACGATGTTAC | gentamicin screening |
| P92 | CTTGCACGTAGATCACATAAGC |  |
