# Supplementary material for: Breaking a barrier: In trans vlsE recombination and genetic manipulation of the native vlsE gene of the Lyme disease pathogen
Source: PLoS Pathog. 2025 Jan 10;21(1):e1012871. doi: 10.1371/journal.ppat.1012871 (PMC11756760; doi:10.1371/journal.ppat.1012871)
Supplement: S2 Table — (DOCX) [file ppat.1012871.s009.docx]

**Table S2- Strains and plasmids used in the study**

| **Strains/plasmids** | **Description** | **Reference** |
| --- | --- | --- |
| B31- A3 | *B. burgdorferi* wild type strain B31 clone A3 | [67] |
| B31-A1 | *B. burgdorferi* strain B31 clone A1 lacking lp28-1 | [67] |
| B31-5A10 | *B. burgdorferi* strain B31 clone 5A10 lacking lp25 and lp56 | [48] |
| pBSV2::*vlsE* | pBSV2 shuttle vector plasmid carrying *vlsE* and *pncA* with their native promoters- circular topology | This study |
| pBSV2*rtel*::*vlsE* | pBSV2 shuttle vector plasmid carrying *vlsE*, *pncA* with their native promoters, and *rtel-* linear topology | This study |
| pBSV2*rtel*::*vlsE:*DR | pBSV2 shuttle vector plasmid harboring mutations in direct repeat regions of *vlsE* with native promoter | This study |
| pBSV2G*::pncA* | pBSV2G shuttle vector plasmid carrying *pncA* with native promoter and lacking *vlsE having gentamicin resistance marker* | This study |
| pBSV2*rtel*::*vlsE:A220** | pBSV2 shuttle vector plasmid harboring mutation in invariable region 4 (IR4) of *vlsE* with native promoter, substituting alanine at position 220 with TAA stop codon | This study |
| pBSV2*rtel*::*vlsE:A282** | pBSV2 shuttle vector plasmid harboring mutation in invariable region 6 (IR6) of *vlsE* with native promoter, substituting alanine at position 282 with TAA stop codon | This study |
